# Reactivity of Acrylamides Causes Cytotoxicity and Activates Oxidative Stress Response

**DOI:** 10.1021/acs.chemrestox.3c00115

**Published:** 2023-08-02

**Authors:** Julia Huchthausen, Beate I. Escher, Nico Grasse, Maria König, Stephan Beil, Luise Henneberger

**Affiliations:** † Department of Cell Toxicology, Helmholtz Centre for Environmental Research − UFZ, Permoserstr. 15, 04318 Leipzig, Germany; ‡ Department of Geosciences, Eberhard Karls University Tübingen, Environmental Toxicology, 72076 Tübingen, Germany; § Department of Analytical Chemistry, Helmholtz Centre for Environmental Research − UFZ, Permoserstr. 15, 04318 Leipzig, Germany; ∥ Institute of Water Chemistry, Technische Universität Dresden, 01069 Dresden, Germany

## Abstract

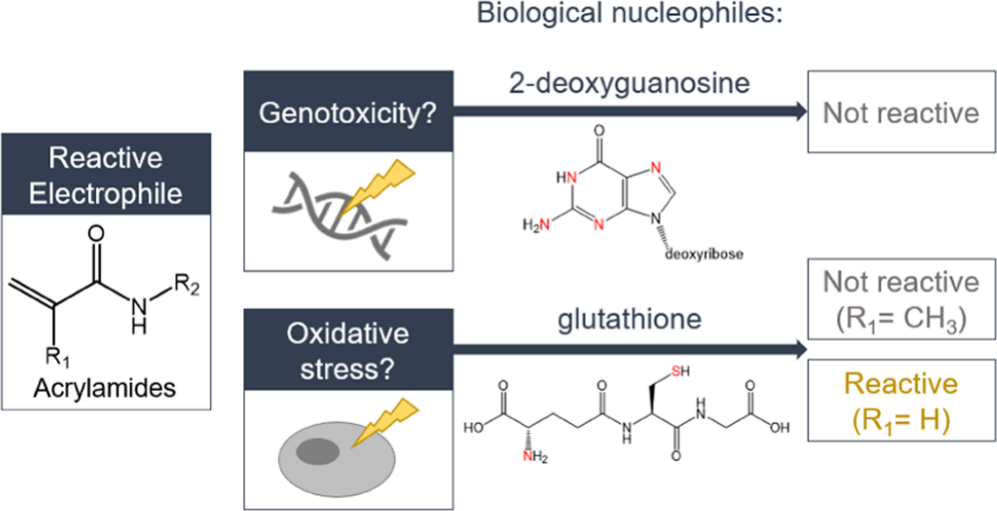

Acrylamides are widely used industrial chemicals that cause adverse effects in humans or animals, such as carcinogenicity or neurotoxicity. The excess toxicity of these reactive electrophilic chemicals is especially interesting, as it is mostly triggered by covalent reactions with biological nucleophiles, such as DNA bases, proteins, or peptides. The cytotoxicity and activation of oxidative stress response of 10 (meth)acrylamides measured in three reporter gene cell lines occurred at similar concentrations. Most acrylamides exhibited high excess toxicity, while methacrylamides acted as baseline toxicants. The (meth)acrylamides showed no reactivity toward the hard biological nucleophile 2-deoxyguanosine (2DG) within 24 h, and only acrylamides reacted with the soft nucleophile glutathione (GSH). Second-order degradation rate constants (*k*_GSH_) were measured for all acrylamides with *N*,*N*′-methylenebis(acrylamide) (NMBA) showing the highest *k*_GSH_ (134.800 M^–1^ h^–1^) and *N*,*N*-diethylacrylamide (NDA) the lowest *k*_GSH_ (2.574 M^–1^ h^–1^). Liquid chromatography coupled to high-resolution mass spectrometry was used to confirm the GSH conjugates of the acrylamides with a double conjugate formed for NMBA. The differences in reactivity between acrylamides and methacrylamides could be explained by the charge density of the carbon atoms because the electron-donating inductive effect of the methyl group of the methacrylamides lowered their electrophilicity and thus their reactivity. The differences in reactivity within the group of acrylamides could be explained by the energy of the lowest unoccupied molecular orbital and steric hindrance. Cytotoxicity and activation of oxidative stress response were linearly correlated with the second-order reaction rate constants of the acrylamides with GSH. The reaction of the acrylamides with GSH is hence not only a detoxification mechanism but also leads to disturbances of the redox balance, making the cells more vulnerable to reactive oxygen species. The reactivity of acrylamides explained the oxidative stress response and cytotoxicity in the cells, and the lack of reactivity of the methacrylamides led to baseline toxicity.

## Introduction

Monomeric acrylamide (prop-2-enamide) is used in the chemical industry for the production of adhesives, sealants, coating products, and inks. Acrylamide is the building block of polyacrylamide, which is widely used in research, water treatment, and papermaking.^1,2^ Acrylamide can also be formed during food processing at high temperatures.^3^ It has been identified as a rodent carcinogen and probable human carcinogen^4,5^ and is known to cause neurotoxicity in humans.^6,7^ This is why, the European Union established a benchmark level for acrylamide in food in 2017.^8^ The toxicity of acrylamide is well studied, and risks are known for this chemical, but the chemical group of acrylamides includes a large number of chemicals with different physicochemical properties and few data are available concerning the toxicity of differently *N*-substituted acrylamides (CH_2_=CHC(O)NR_2_) and methacrylamides (CH_2_=C(CH_3_)C(O)NR_2_) even though some of these chemicals are produced in large quantities and also find application in industry and research.^9,10^

**Figure 1:**
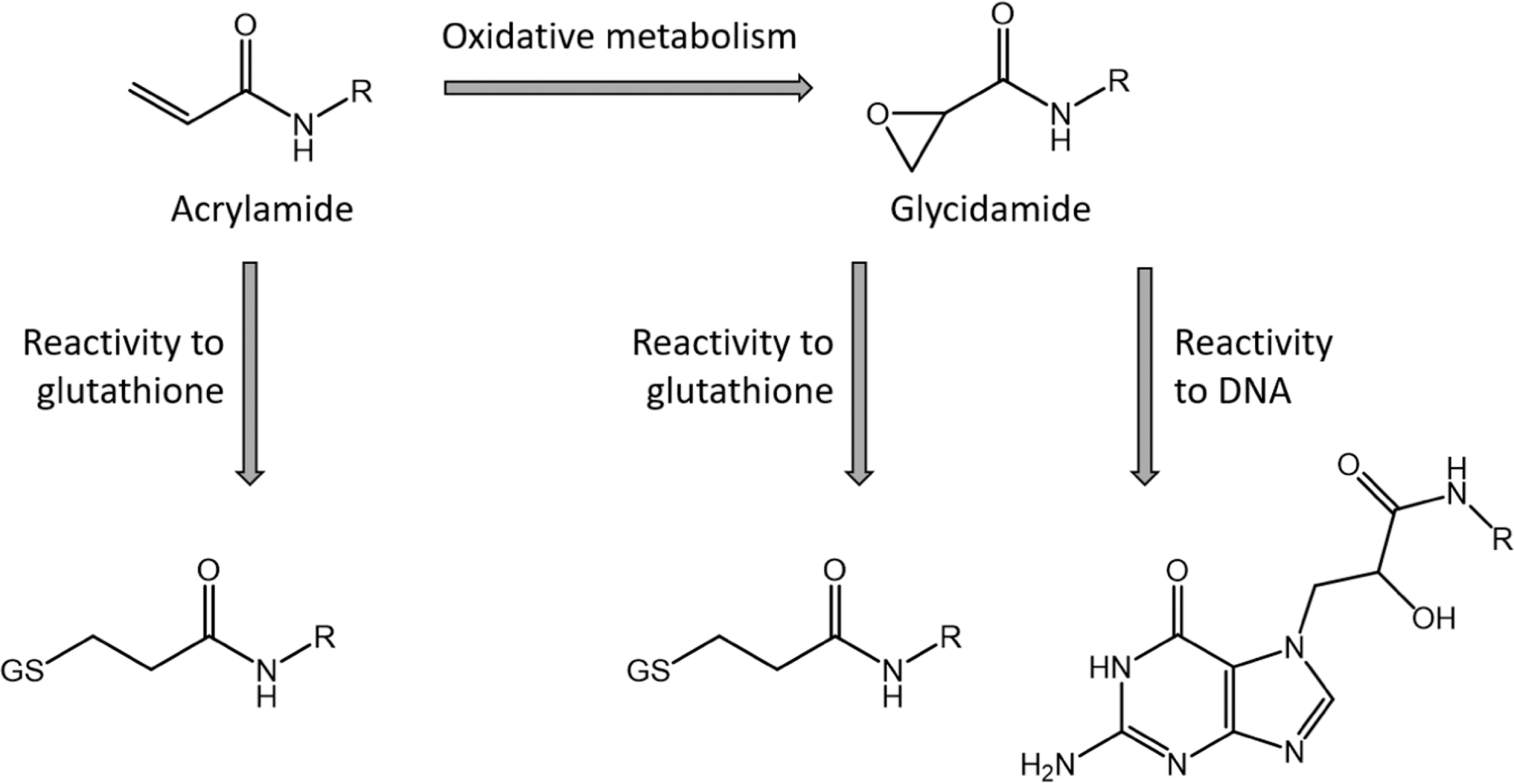
Potential reaction pathways of acrylamide chemicals in in vitro bioassays. Adapted from Katen et al.^28^

Acrylamides belong to the group of electrophilic reactive chemicals. The toxicity of reactive chemicals exceeds baseline toxicity (narcosis),^11^ which is the lowest toxicity a chemical can have and is caused by the incorporation of the chemicals into the cell membrane.^12^ Reactive chemicals are of special concern since they usually have 10 to 10,000 times higher toxicity than baseline toxic chemicals,^11^ and their toxicity can have different modes of action (MOA),^13^ but is mostly triggered by irreversible reactions with thiol, amino, or hydroxyl groups of biological nucleophiles such as proteins, peptides, and DNA.^14−16^ The reaction of acrylamides with nucleophiles is a Michael addition where the α,β-unsaturated carbonyl moiety of the acrylamide acts as the Michael acceptor and the biological nucleophile acts as the Michael donor.^17,18^ Especially the tripeptide glutathione (γ-l-glutamyl-l-cysteinyl-glycin, GSH) is a target of reactive chemicals since it has a free thiol group and is present in large amounts in the cell (Figure 1).^19^ Acrylamide can also be metabolized by cytochrome P450 2E1 to its metabolite glycidamide, which reacts with DNA bases and causes genotoxicity (Figure 1).^20,21^ All of these processes play a role in the toxicity of reactive chemicals and may lead to a variety of adverse effects of these chemicals.

Even though acrylamides are reactive chemicals and their reactivity is unspecific, they do not react equally well with all biological nucleophiles. For example, acrylamide reacts very rapidly with the thiol GSH but requires metabolic activation to react with DNA.^22,23^ The selectivity in the reaction between electrophilic and nucleophilic chemicals can be explained by Pearson’s theory of hard and soft acids and bases (HSAB).^24^ According to this concept, reactive molecules are classified based on their respective polarizabilities as either soft (polarizable) or hard (nonpolarizable) electrophiles or nucleophiles. Furthermore, chemicals with the same softness or hardness react preferentially with each other.^25,26^ The polarizability of a molecule depends on its electron distribution. The conjugated α,β-unsaturated carbonyl structure of acrylamides is a soft electrophile because of the delocalized pi-electron system. Therefore, they react preferentially with soft nucleophiles, such as thiols, which are easily polarized due to the large atomic radius of sulfur. The nitrogen and oxygen nucleophiles in DNA or RNA have smaller atomic radii and thus represent harder nucleophiles, which react preferentially with hard electrophiles, such as epoxides or organochlorides.^25,27^ Quantum chemical calculations can be used to rationalize the chemical reactivity. The lowest unoccupied molecular orbital (LUMO) of the electrophile and the highest occupied molecular orbital (HOMO) of the nucleophile determine the reaction rate. The selectivity of the reaction can be modeled by the energies of these orbitals. Hard electrophiles usually have a relatively high energy (ε) of the LUMO (ε_LUMO_) and soft electrophiles have rather low or negative ε_LUMO_. However, other molecular structures can also play a role in reactivity if they sterically hinder the reaction.^26,27^

The determination of the molecular target of reactive chemicals is important for the assessment of their toxicity and for the identification of possible adverse outcomes in humans.^16,29^ Therefore, various studies have focused on the relationship between the reactivity of chemicals and toxicity in different species, such as bacteria, ciliophoran, algae, and fish.^30−34^

**Figure 2:**
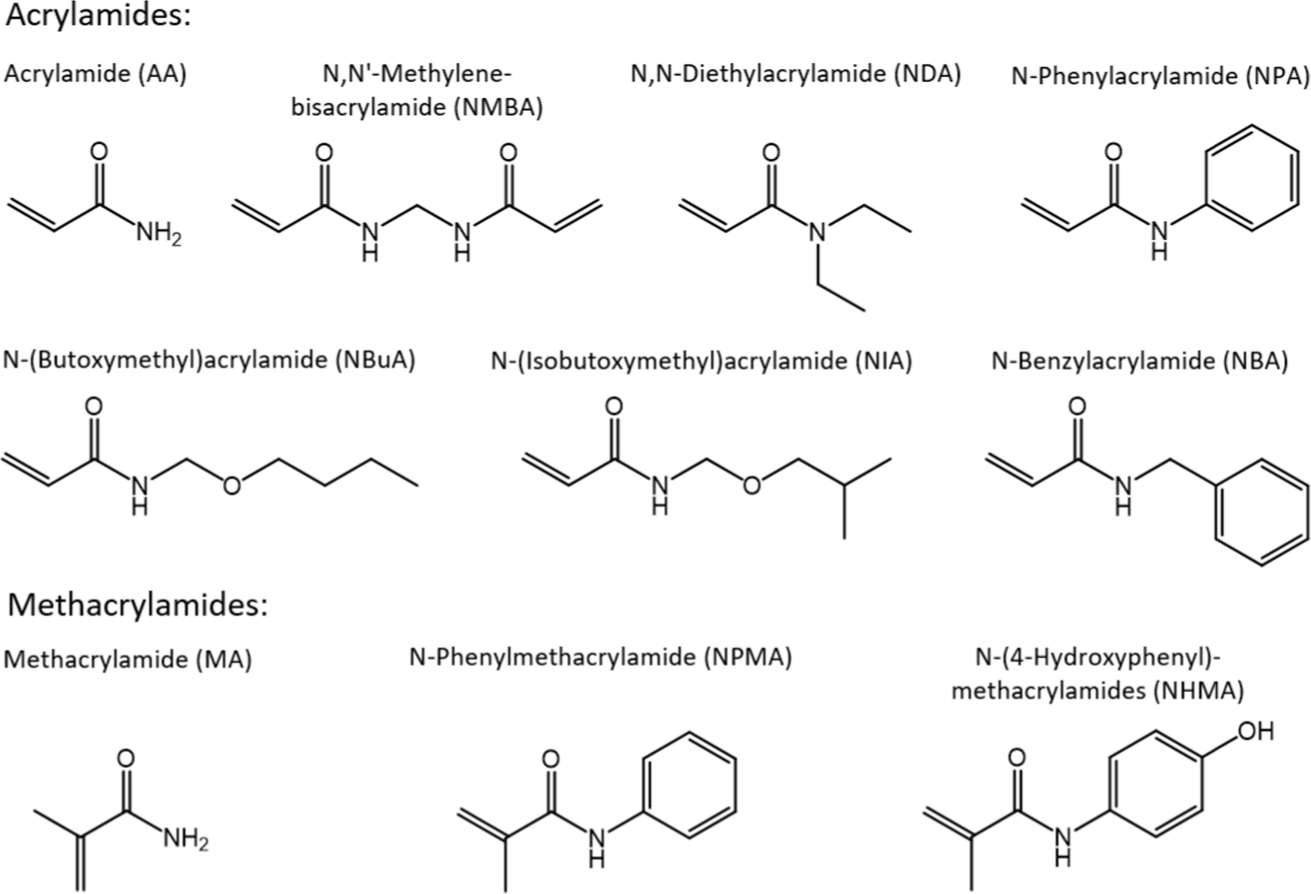
Structures of the test chemicals.

The aim of this study was to investigate whether the *in vitro* toxicity of different substituted (meth)acrylamides depends on their reactivity toward biological nucleophiles and whether this reactivity can be explained by their chemical structure. There are major knowledge gaps regarding the toxicity of substituted (meth)acrylamides, but these chemicals are used in large quantities in industry and may pose a potential hazard to humans and the environment. For this study, we selected (meth)acrylamides with different physicochemical properties that are produced in large quantities to investigate a possible influence of the substituent on reactivity and toxicity. The final test set of chemicals consisted of one bipolarized compound, two primary amines, six secondary amines, and one tertiary amine with seven chemicals being acrylamides and three being methacrylamides (Figure 2). The hydrophobicity of the test chemicals ranged over 2 orders of magnitude (Table S1). The cytotoxicity of the test chemicals was measured in three reporter gene cell lines (GR-*bla*, ARE-*bla*, and AREc32). GR-*bla* is based on a HEK293T cell line, ARE-*bla* is based on a HepG2 cell line, and AREc32 is based on an MCF7 cell line.

Previous work has demonstrated that GR-*bla* has no cytochrome P450 activity, while ARE-*bla* has a higher basal CYP1 level and CYP1 is inducible by chemical exposure in AREc32.^35^ Three cell lines with different metabolic capacities were chosen to relate possible differences in cytotoxicity to differences in metabolic activity since it has been shown that metabolic activation of acrylamide to the reactive glycidamide is necessary for the reaction with DNA.^36^

ARE-*bla* and AREc32 also carry a reporter gene for the antioxidant response element, allowing measurement of the oxidative stress response via the Nuclear Factor Erythroid 2-related Factor 2/Kelch-like ECH-associated protein 1 (Nrf-2/Keap-1) pathway. This metabolic pathway is mostly activated by the generation of reactive oxygen species (ROS) in the exposed cells, but for some reactive chemicals, the oxidative stress response can also be triggered by direct binding of the chemicals to Keap-1.^37,38^ The end point can also be an indirect measure of the reaction of the test chemicals with GSH, which maintains the redox status of the cells. GSH also functions as a detoxification molecule, as a deficiency of GSH leads to reduced protection against ROS, which can ultimately lead to cell death.^39^ Since acrylamides, as soft electrophiles, react preferentially with soft nucleophiles such as GSH or cysteine residues in cellular proteins, two assays were selected that reflect this MOA. Direct reactions with DNA were not expected^40^ and hence no assay for genotoxicity was selected. GR-*bla* carries a reporter gene for glucocorticoid receptor, which is not of interest for reactive chemicals^41^ and only the cytotoxicity was quantified for this cell line.

Reactivity toward the hard nucleophile 2-deoxyguanosine (2DG) was investigated to confirm our hypothesis that reaction with DNA is not the molecular initiating event. Degradation rates and half-lives of the test chemicals toward the soft biological nucleophile GSH were measured and compared to the toxicity and activation of the Nrf-2/Keap-1 pathway and used to derive information on the MOA of the test chemicals. The reactivity of acrylamides with GSH has been previously described,^36,42^ but we expanded systematically to acrylamides and methacrylamides with substitute groups to investigate how the substitution affects the toxicity and reactivity of the chemicals. In addition, we used nontarget screening to identify the GSH conjugates of acrylamides and quantum chemical calculations to explain the reactivity of the chemicals to GSH.

## Materials and Methods

### Chemicals

The chemicals acrylamide (79-06-1, AA), *N,N*′-methylenebis(acrylamide) (110-26-9, NMBA), *N*-(butoxymethyl)acrylamide (1852-16-0, NBuA), *N*-(isobutoxymethyl)acrylamide (16669-59-3, NIA), *N,N*-diethylacrylamide (2675-94-7, NDA), methacrylamide (79-39-0, MA), *N*-benzylacrylamide (13304-62-6, NBA), *N*-phenylmethacrylamide (1611-83-2, NPMA), *N*-phenylacrylamide (2210-24-4, NPA), and *N*-(4-hydroxyphenyl)methacrylamide (19243-95-9, NHMA) were used in this study. Chemical structures are shown in Figure 2, and more information about the test chemicals can be found in the Supporting Information (Table S1).

### Materials

All components of the bioassay media and GeneBLAzer ARE-*bla* and GeneBLAzer GR-UAS-*bla* cells were purchased from Thermo Fisher Scientific. AREc32 cells^43^ were obtained from Cancer Research UK. 2′-Deoxyguanosine monohydrate (Cayman Chemical; Cay9002864-5; 312693-72-4) and reduced glutathione (Sigma-Aldrich; G4251-5G; 70-18-8) had a purity of ≥98%. All solvents used were of LC-MS grade and had a purity of ≥99%. Acetonitrile and 2-propanol were purchased from Honeywell or Chemsolute. Methanol was purchased from Honeywell, and formic acid was purchased from Serva. Water was obtained from a Milli-Q water purification system from Merck. Supel BioSPME 96-Pin Devices (Sigma-Aldrich; 59683-U) coated with C18-particles embedded in polyacrylonitrile (PAN) were used. The coating length was 2.1 mm, and the average coating thickness was 12.5 μm, resulting in an approximate coating volume of 80 nL.^44,45^ Polystyrene 384-well plates (Product Nos. 3765 and 356663) from Corning were used for the *in vitro* bioassays, and the reactivity tests were performed in glass-coated deep-well plates (Product No. 60180P336) from Thermo Fisher Scientific which were sealed with sealing film from Brand (Product No. 701367).

### *In Vitro* Bioassay

ARE-*bla* bioassay medium (90% DMEM with GlutaMAX phenol red-free, 10% dialyzed fetal bovine serum (FBS), 0.1 mM nonessential amino acids, 25 mM HEPES, 100 U/mL penicillin-streptomycin), GR-*bla* bioassay medium (98% Opti-MEM, 2% charcoal-stripped FBS, 100 U/mL penicillin-streptomycin), and AREc32 bioassay medium (90% DMEM with GlutaMAX, 10% FBS, 100 U/mL penicillin-streptomycin) were used. A detailed description of the bioassay procedure can be found in the literature.^46−48^

Briefly, 30 μL of cell suspension in assay medium was dispensed into each well of a poly-d-lysine treated black 384-well plate with clear bottom (Product No. 356663, ARE-*bla* and GR-*bla*) or a white 384-well plate with clear bottom (Product No. 3765, AREc32) using a MultiFlo Dispenser (Biotek, Vermont, USA). The final cell numbers were 4100 cells/well (ARE-*bla*), 6000 cells/well (GR-*bla*) and 2650 cells/well (AREc32). The plates were incubated at 37 °C and 5% CO_2_ for 24 h, and the confluency of the cells was measured with an IncuCyte S3 Live-Cell Analysis System (Essen BioScience, Sartorius) before and 24 h after chemical dosing. Chemical dilutions in the respective bioassay media were prepared by dissolving the pure chemical directly in the medium (AA, NMBA, NBuA, NIA, NDA, MA) or by using stock solutions in methanol (NBA, NPMA, NPA, NHMA). The final methanol content in the well was kept below 1%. All chemicals were tested in all assays in three independent replicates in 11-step serial dilutions. Dosing plates containing the chemicals in serial dilution were prepared using a Hamilton Microlab Star robotic system (Hamilton, Bonaduz, Switzerland). The diluted chemicals were dosed in duplicates by transferring two times 10 μL from the dosing plates to the cell plate. The cell plates were incubated at 37 °C and 5% CO_2_ for 24 h. The cytotoxicity was evaluated by comparing the relative confluency of the cells before and after dosing. The activation of the reporter genes was quantified as described in the literature.^46−48^

### Solid-Phase Microextraction

A previously published high-throughput (HT) solid-phase microextraction (SPME) method^45^ was used to extract the chemicals from the medium samples and reaction solutions. The method was automated using a Hamilton Microlab Star robotic system (Hamilton, Bonaduz, Switzerland) equipped with a CO-RE grip and iSWAP and two BioShake 3000-T elm (QInstruments, Jena, Germany) and the corresponding software Hamilton Run Control and Hamilton Method Editor (version 4.5.0.7977). More information about the experimental parameters and a depiction of the robot deck layout can be found in the Supporting Information (Table S2 and Figure S1). The pin device was positioned in an empty deep-well reservoir equipped with a customized metal frame in the Hamilton robot. The remaining labware was also positioned as described in Figure S1. The pin device was conditioned in isopropanol for 20 min, in Milli-Q water for 10 s, and then transported to the deep-well plate containing the sample solutions. The chemicals were extracted at 1000 rpm and 37 °C for 15 min, then the pin device was transferred to the desorption plate containing the respective desorption solutions (Table S1) and was desorbed at 1000 rpm and room temperature for 15 min. No wash was performed between the extraction and desorption. Finally, the pin device was transported back to its starting position. All desorption plates were sealed and stored at 4 °C until concentration measurement.

### Stability in Assay Medium

The freely dissolved concentration (*C*_free_) of the test chemicals was measured in ARE-*bla* bioassay medium, GR-*bla* bioassay medium, and AREc32 bioassay medium. Chemical stock solutions of the test chemicals were spiked into aliquots of the media at a final concentration of 5.0 × 10^–4^ M (AA, NMBA, and MA) or 3.0 × 10^–4^ M (NBuA, NIA, NDA, NBA, NPMA, NPA, NHMA). 600 μL of each reaction solution were transferred in duplicate into two glass-coated 96-deep-well plates. One of the plates was directly extracted using SPME. The other plate was incubated at 37 °C for 24 h before extraction.

### Reactivity Testing

To determine the reactivity of the test chemicals, reduced glutathione (GSH) and 2′-deoxyguanosine (2DG) were dissolved in phosphate-buffered saline (PBS, 137 mM NaCl, 12 mM phosphate) at different concentrations, and the pH was adjusted to 7.4. The test chemicals were added to aliquots of the GSH or 2DG solutions, leading to the same final concentrations as described above for the medium samples. The concentration of GSH was the same, 2 times, 5 times, 10 times, 50 times, or 100 times higher than the chemical concentration. The concentration of 2DG was the same, two times, five times, 10 times, 20 times, or 30 times higher than the chemical concentration. 600 μL of each reaction solution were transferred in duplicate into seven glass-coated 96-deep-well plates. One of the plates was directly extracted using SPME. The other plates were incubated at 37 °C for 30 min and 1, 2, 4, 6, or 24 h before extraction. The experiments were performed three times for all chemicals and solutions if the chemicals were degraded in the first test.

### Instrumental Analysis

The chemical concentration in the desorption solvent was measured using a liquid chromatography instrument (LC, Agilent 1260 Infinity II) coupled to a triple quadrupole mass spectrometer (MS, Agilent 6420 Triple Quad). A LunaOmega 1.6 μm, Polar C18, 100 Å, LC column (50 × 2.1 mm) was used for AA, NMBA, NBuA, NIA, NDA, and MA. A Kinetex 1.7 μm, C18, 100 Å, LC column (50 × 2.1 mm) was used for NBA, NPMA, NPA, and NHMA. All LC and MS parameters can be found in the Supporting Information (Table S3). Standard solutions in the respective desorption solvents (1–5000 ng/L) and acetonitrile blanks were measured together with the samples.

One replicate of the desorption solvents after SPME of the chemicals incubated with GSH (ratio GSH/acrylamide = 5:1 and 100:1) for 1, 4, and 24 h was transferred to HPLC vials with inserts and analyzed by ultraperformance liquid chromatography time-of-flight mass spectrometry (UPLC-TOF-MS) using a AQUITY UPLC I-Class system (Waters) equipped with a HSS T3 column (100 mm × 2.1 mm, 1.7 μm) coupled to a XEVO XS Q-TOF-MS (Waters) to identify conjugates of the reaction of the acrylamides with GSH. The samples were injected without further dilution and solvent blanks, control samples without the test chemical, as well as control samples without GSH were measured in parallel. A detailed description of the analytical method and the instrumental parameters can be found in the literature.^49^ GSH adducts were detected by a screening approach using MarkerLynx (Waters, version 4.1). UPLC-MS data were evaluated in a retention time window of 1 to 10 min and a mass range of *m*/*z* 50–1200. The maximum deviation in retention time for peak picking was 0.1 min, and the maximum deviation in the exact mass was 0.01 Da. Peaks that were present only in the samples containing acrylamide and GSH and not in the solvent blanks and control samples of acrylamides without GSH were selected as candidate conjugates. Chemical formulas were generated using a mass tolerance of 5 ppm and elemental composition of C (0–100), H (0–100), N (0–20), O (0–20), S (0–20), and Na (0–2). Additionally, fragment ions were considered for the structure elucidation.

### Data Evaluation

An automatic KNIME (version 4.6.1) workflow and GraphPad Prism (version 9.0.2) were used for the evaluation of the bioassay data. The measured cytotoxicity and effects were plotted against the chemical concentration in the linear range of the concentration–response curve and the inhibitory effect concentrations were derived from the slope of the regression.^50^

The IC_10_ for cytotoxicity is the concentration at which a reduction in cell viability of 10% is achieved and was calculated with eq 1.
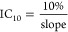
1For the evaluation of the activation of the oxidative stress response, the induction ratio (IR) was calculated and the EC_IR1.5_ (eq 2) was derived from the slope of the concentration–response curve.^48^
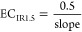
2The reference substances used were dexamethasone for GR-*bla* and *tert*-butylhydroquinone (tBHQ) for AREc32 and ARE-*bla*.

The IC_10,baseline_ was calculated with the baseline quantitative structure–activity relationship (QSAR) from Lee et al. (2021),^51^ where *K*_lip/w_ stands for the liposome-water partition constant, a measure of hydrophobicity and affinity to biological membranes. *K*_lip/w_ of all test chemicals were predicted using a linear solvation energy relationship (LSER) model (Table S1).^52^
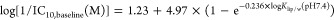
3To compare the measured cytotoxicity IC_10_ with the baseline toxicity IC_10,baseline_, the toxic ratio (TR) was calculated with eq 4.
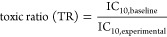
4The specificity ratio (SR) was used to elucidate how much the reporter gene induction differs from cytotoxicity (SR_cytotoxicity_, eq 5) or baseline toxicity (SR_baseline_, eq 6).^53^
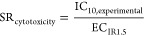
5
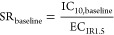
6The freely dissolved concentrations of the chemicals in the bioassay medium (*C*_free_) were calculated with eq 7 as a measure of the exposure concentration.^54^ The total amount of chemicals in the medium (*n*_total_) was calculated from the nominal concentration (*C*_nom_) added to the medium. The pin-water distribution ratios (*D*_pin/w_) were calculated from samples in phosphate-buffered saline (PBS) (see the Supporting Information Text S1 for more information). Chemical concentrations in the pin coating (*C*_pin_) were calculated from the measured concentrations in the desorption solution after SPME and the volume of the pin coating (*V*_pin_) was approximately 80 nL.
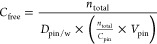
7To determine the first-order degradation rate constant (*k*) and the degradation half-life (*t*_1/2_) of the chemicals, the natural logarithm (ln) of the chemical concentration in the desorption solvent after SPME (*C*_des_) was plotted against the incubation time (*t*). From the linear regression of this plot, *k* could be derived (eq 8).
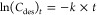
8The degradation half-life (*t*_1/2_) was calculated from *k* using eq 9.
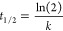
9The second-order rate constant from the reaction of acrylamides with GSH (*k*_GSH_) was determined by linear regression of *k* plotted against the concentration of GSH (eq 10), where *k*_GSH_ is the slope of the regression and the intercept is *k*_H_2_O_, the reaction rate constant of the reaction with water.
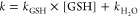
10

### Quantum Chemical Calculations

Charge densities of selected atoms (*q*(C_α_), *q*(C_β_), and *q*(C_1_)) and the energy of the lowest unoccupied orbital (ε_LUMO_) were calculated for all test chemicals. 3D structure files of acrylamides were generated using Avogadro software, version 1.2.0,^55^ and initially geometry-optimized via steepest descent algorithm in the UFF force field.^56^ The resulting Cartesian coordinates were used as input for a detailed MP2 calculation (second-order Møller–Plesset perturbation theory) with the def2-TZVP basis set. The calculations were run with ORCA software, version 5.0.3.^57−59^ Subsequently, the same software was applied to convert the obtained files to the molden file format. Orbitals of the final structures were visualized via IboView software, version 20211019-RevA.^60^

## Results and Discussion

### Cytotoxicity

The measured cytotoxicity IC_10_ values (Table 1) were derived from the concentration–response curves shown in the Supporting Information (Figures S2–S4). NMBA was the most cytotoxic chemical, with the lowest IC_10_ values in all assays. The comparison of the IC_10_ values from ARE-*bla* and GR-*bla* with those from AREc32 showed that the measured cytotoxicity of the chemicals in the assays differed by less than 1 order of magnitude (Figure S5). The similar toxicity of the chemicals in cell lines of different origins suggests that differences in the metabolic activity of the cells do not affect the toxicity of the chemicals.

**Table 1:** IC_10_ and EC_IR1.5_ Values for All Chemicals and Assays^a^

	ARE-*bla*				AREc32				GR-*bla*	
chemical	IC_10_ [M]	CV [%]	EC_IR1.5_ (M)	CV [%]	IC_10_ (M)	CV [%]	EC_IR1.5_ (M)	CV [%]	IC_10_ (M)	CV [%]
AA	1.01 × 10^–3^	26.1	4.34 × 10^–4^	7.2	4.29 × 10^–4^	7.0	3.10 × 10^–4^	5.9	8.05 × 10^–4^	4.9
NMBA	1.13 × 10^–4^	16.2	1.54 × 10^–5^	7.0	4.25 × 10^–5^	11.3	1.16 × 10^–5^	4.6	8.29 × 10^–5^	7.2
NBuA	5.28 × 10^–4^	10.0	4.96 × 10^–5^	7.6	1.52 × 10^–4^	7.7	5.43 × 10^–5^	4.8	3.90 × 10^–4^	5.3
NIA	8.74 × 10^–4^	8.8	6.76 × 10^–5^	7.5	2.74 × 10^–4^	6.0	8.62 × 10^–5^	5.9	5.34 × 10^–4^	8.2
NDA	8.06 × 10^–3^	8.5	6.08 × 10^–4^	6.1	5.73 × 10^–3^	6.1	6.89 × 10^–4^	3.0	3.10 × 10^–3^	5.3
MA	2.23 × 10^–2^	14.9	4.28 × 10^–3^	3.9	1.59 × 10^–2^	13.4	2.53 × 10^–3^	4.6	7.56 × 10^–3^	6.4
NBA	2.96 × 10^–3^	6.7	2.92 × 10^–4^	8.3	2.33 × 10^–3^	17.2	2.61 × 10^–4^	2.3	1.44 × 10^–3^	12.1
NPMA	6.20 × 10^–3^	22.1	3.27 × 10^–3^	10.3	2.66 × 10^–3^	12.6	1.33 × 10^–3^	7.5	1.42 × 10^–3^	9.0
NPA	7.58 × 10^–4^	6.8	4.01 × 10^–5^	7.5	2.12 × 10^–4^	9.2	6.00 × 10^–5^	4.3	4.35 × 10^–4^	9.9
NHMA	5.41 × 10^–2^	34.7	7.55 × 10^–3^	6.6	1.19 × 10^–2^	21.2	3.88 × 10^–3^	6.0	1.30 × 10^–3^	6.7
tBHQ	>1.73 × 10^–5^		2.93 × 10^–6^	3.4	>1.73 × 10^–5^		2.91 × 10^–6^	2.0	not tested	

a Chemical structures are shown in Figure 2. CV represents the coefficient of variation based on three independent replicates.

### Activation of Oxidative Stress Response

Oxidative stress response was activated by all chemicals in the ARE-*bla* and the AREc32 assay (Table 1 and Figures S2 and S4), which is in line with previous studies which identified acrylamide as an activator of oxidative stress response *in vitro*(61,62) and *in vivo*.^63^ NMBA showed the strongest effect in both assays.

The comparison of the measured EC_IR1.5_ values in ARE-*bla* and AREc32 also showed almost perfect agreement. This suggests that the activation of ARE of the chemicals is independent of cell type and origin, so metabolic activation is not necessary to trigger the effect and the MOA is the same in different cell types.

### Specificity Analysis

**Figure 3:**
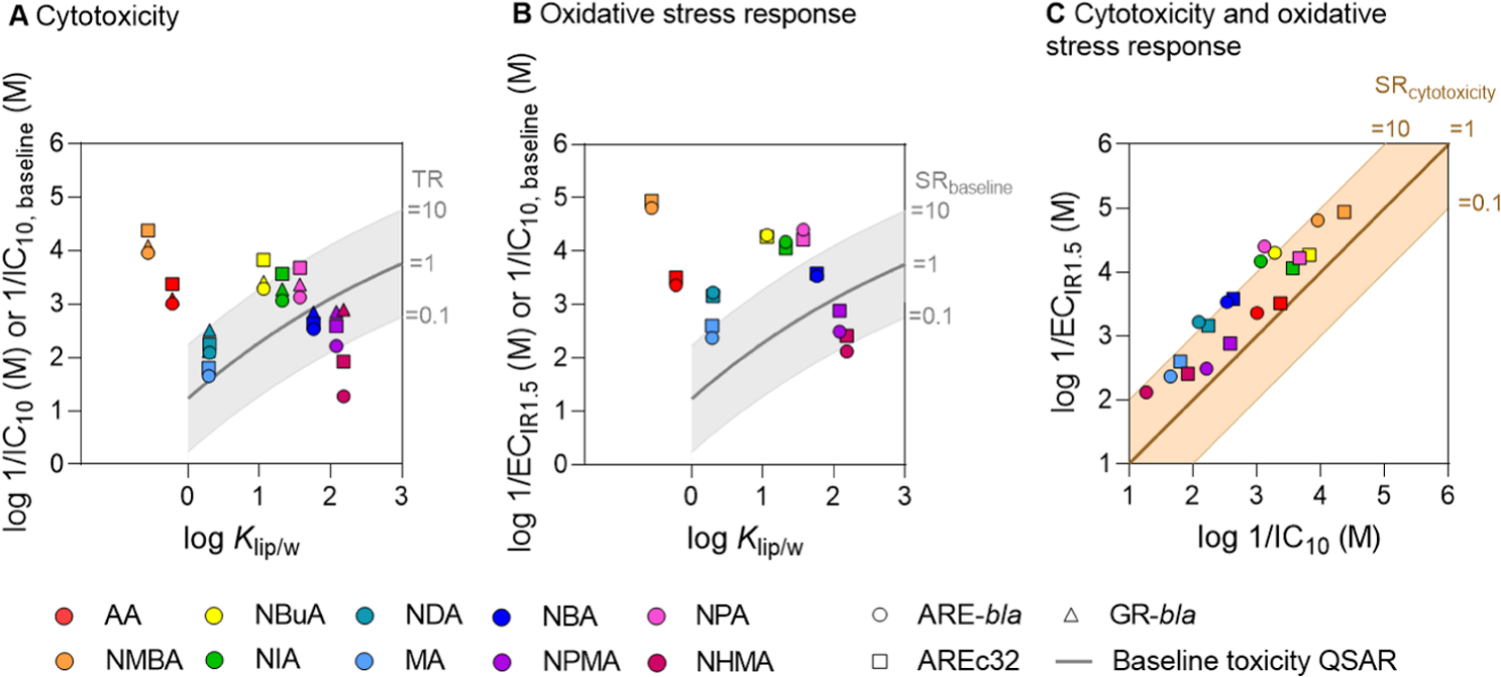
Visualization of toxic ratios (TR) and specificity ratios (SR) of the test chemicals. The thick gray lines in (A) and (B) represent the predicted baseline IC_10_ (eq 4) as a function of the logarithmic liposome-water partition constant log *K*_lip/w_ for chemicals with *K*_lip/w_ ≥ 0.^51^ (A) Cytotoxicity (log 1/IC_10_) plotted against log *K*_lip/w_. Gray areas show TR values between 0.1 and 10. (B) Activation of the oxidative stress response (log 1/EC_IR1.5_) plotted against log *K*_lip/w_. Gray areas show SR_baseline_ values between 0.1 and 10. (C) Activation of the oxidative stress response (log 1/EC_IR1.5_) plotted against cytotoxicity (log 1/IC_10_). The thick brown line indicates an SR_cytotoxicity_ of 1, and the brown area shows SR_cytotoxicity_ values between 0.1 and 10. The different symbols indicate three different assays.

In Figure 3, the cytotoxicity log 1/IC_10_ (Figure 3A) and the activation of the oxidative stress response log 1/EC_IR1.5_ (Figure 3B) are plotted against the hydrophobicity (*K*_lip/w_) of the test chemical. There was no apparent relationship between log *K*_lip/w_ and cytotoxicity or activation of the oxidative stress response. Additionally, the baseline toxicity QSAR^51^ was plotted as a function of the *K*_lip/w_ to visualize the toxic ratios (TR) and specificity ratios (SR_baseline_) of the chemicals, showing the comparison of the measured effects, namely, IC_10_ and EC_IR1.5_, with the predicted IC_10,baseline_. Baseline toxicity is the lowest toxicity a substance can have and is triggered by the incorporation of the chemical into the cell membrane.^11,12^ The baseline toxicity QSAR is not defined at log *K*_lip/w_ below 0, because no experimental data were recorded. In addition, very hydrophilic chemicals do not tend to be incorporated into the cell membrane and, thus, are unlikely to act through this mode of action. Unsubstituted acrylamide, like all small and polar molecules, does not accumulate in the cell membrane but rapidly permeates it.^64^ Therefore, it cannot reach the critical membrane concentrations required to trigger baseline toxicity but must cause its toxicity through another mechanism. As hydrophobicity is affected by substitution, methacrylamides and acrylamides with long side chains have a higher log *K*_lip/w_ and thus a greater tendency to integrate into the cell membrane.

Two of the chemicals (AA and NMBA) were too hydrophilic for a TR to be calculated. However, both showed high cytotoxicity in all assays (AA: log 1/IC_10_ up to 3.37, and NMBA: log 1/IC_10_ up to 4.37). Chemicals with TR > 10 were classified as reactive or specifically acting.^11,65^ Four of the chemicals with log *K*_lip/w_ > 0 (NBuA, NIA, NDA, and NPA) showed TR between 1 and 10 in all assays, which is why they can be classified as reactive toxicants.^11,17^ However, TR were not orders of magnitude higher than baseline toxicity but close to the threshold. MA, NBA, NPMA, and NHMA showed TR around 1 and were classified as baseline toxicants.^11,17^

The SR_baseline_ for most of the chemicals was higher than 10 indicating a specific mode of action. Only NPMA and NHMA had an SR_baseline_ around 1, and activation of oxidative stress response can be considered as a result of the cytotoxicity burst phenomenon.^53,66^Figure 3C shows log 1/EC_IR1.5_ plotted against log 1/IC_10_ for the ARE-*bla* and AREc32 assay. This comparison displays the SR_cytotoxicity_, which was between 1 and 10 for most chemicals. The activation of the oxidative stress response and cytotoxicity appear to be linked and do not occur independently of each other in both cell lines.

### Solid-Phase Microextraction

The time until 95% equilibrium was reached (*t*_95%_), recovery, and logarithmic pin-water distribution ratios (log *D*_pin/w_) were quantified for all test chemicals (Figure S6 and Table S4). The *t*_95%_ was below 15 min for all test chemicals and, for most, below 5 min. The recovery was between 86% (NDA) and 118% (NPA). Log *D*_pin/w_ at equilibrium was between 0.61 (MA) and 1.48 (NPA), with *D*_pin/w_ increasing with the hydrophobicity of the test chemicals.

### Stability in Assay Medium

**Figure 4:**
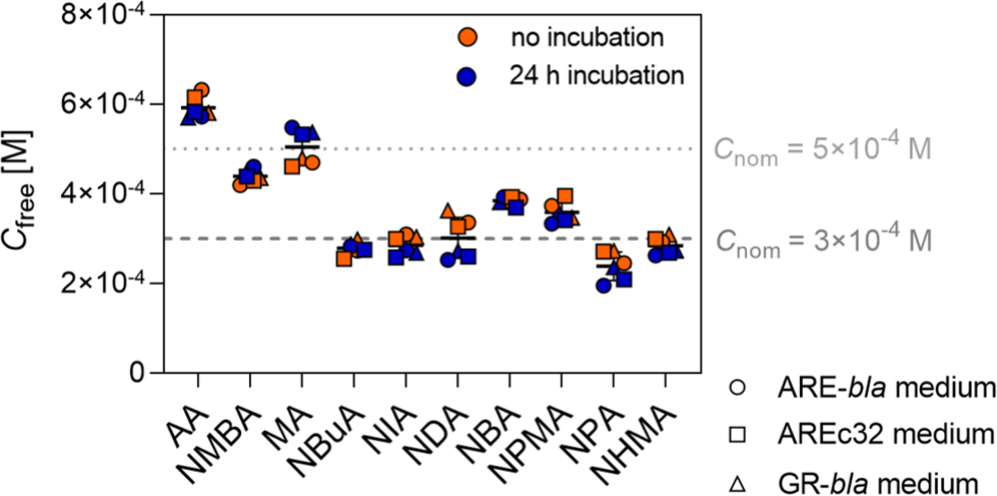
Freely dissolved concentration (*C*_free_) of the test chemicals in three bioassay media without incubation (orange) or after 24 h of incubation (blue). Different symbols represent different media. AA, NMBA, and MA were dosed at a nominal concentration (*C*_nom_) of 5 × 10^–4^ M (gray dotted line), and the other test chemicals were dosed at *C*_nom_ of 3 × 10^–4^ M (dark gray dashed line).

The freely dissolved concentration (*C*_free_) of the test chemicals in GR-*bla*, ARE-*bla*, and AREc32 assay medium was determined without incubation and after 24 h of incubation at 37 °C (Figure 4). The *C*_free_ values of all chemicals were very close (up to a factor of 1.5) to the nominal concentration (*C*_nom_). There was no difference between the *C*_free_ values of the chemicals in the three bioassay media. This is in line with previous results of the measured *C*_free_ of hydrophilic chemicals in *in vitro* bioassays, which showed no or very weak partitioning to proteins in the medium and are therefore almost completely freely dissolved.^54^

For most of the chemicals, there was no difference between *C*_free_ without incubation and *C*_free_ after 24 h, so most chemicals seem to be stable in the bioassay media over 24 h. Only for NDA there was a decrease of *C*_free_ within 24 h of 25%, in the GR-*bla* and ARE-*bla* media and 20% in the AREc32 medium. For NPA, there was a decrease of *C*_free_ of 20% in the ARE-*bla* and 23% in the AREc32 medium. This loss could be caused by covalent reactions of the chemicals with components of the medium since the chemicals showed no degradation over 24 h in aqueous buffer (PBS, pH 7.4) (Figures S7–S10). However, the observed loss of <30% may also be due to experimental variations or measurement uncertainties. This means that the test chemicals do not react or only react slowly with the components of the medium and the concentration is stable for the duration of the bioassay. The proteins in the medium are mostly from fetal bovine serum (FBS), and the results are consistent with previously reported slow reaction of acrylamides and albumin.^42^

### Chemical Reactivity

The test chemicals showed no degradation with 2DG with *t*_1/2_ close to or higher than 50 h (Figures S7 and S8). This value was defined as a threshold, as it is approximately twice the longest incubation time (24 h). To determine more reliable *t*_1/2_ for the reaction with 2DG, longer incubation times would be necessary, but these would not be biologically relevant, since reactive chemicals are rapidly degraded in the body.^40,67^ These results are consistent with the literature as acrylamides show no or only very low reactivity with hard nucleophiles such as DNA bases.^14,22^ Only metabolic activation to more reactive glycidamide enables a reaction with DNA and therefore also causes the mutagenicity of acrylamides.^20,21,68^

**Figure 5:**
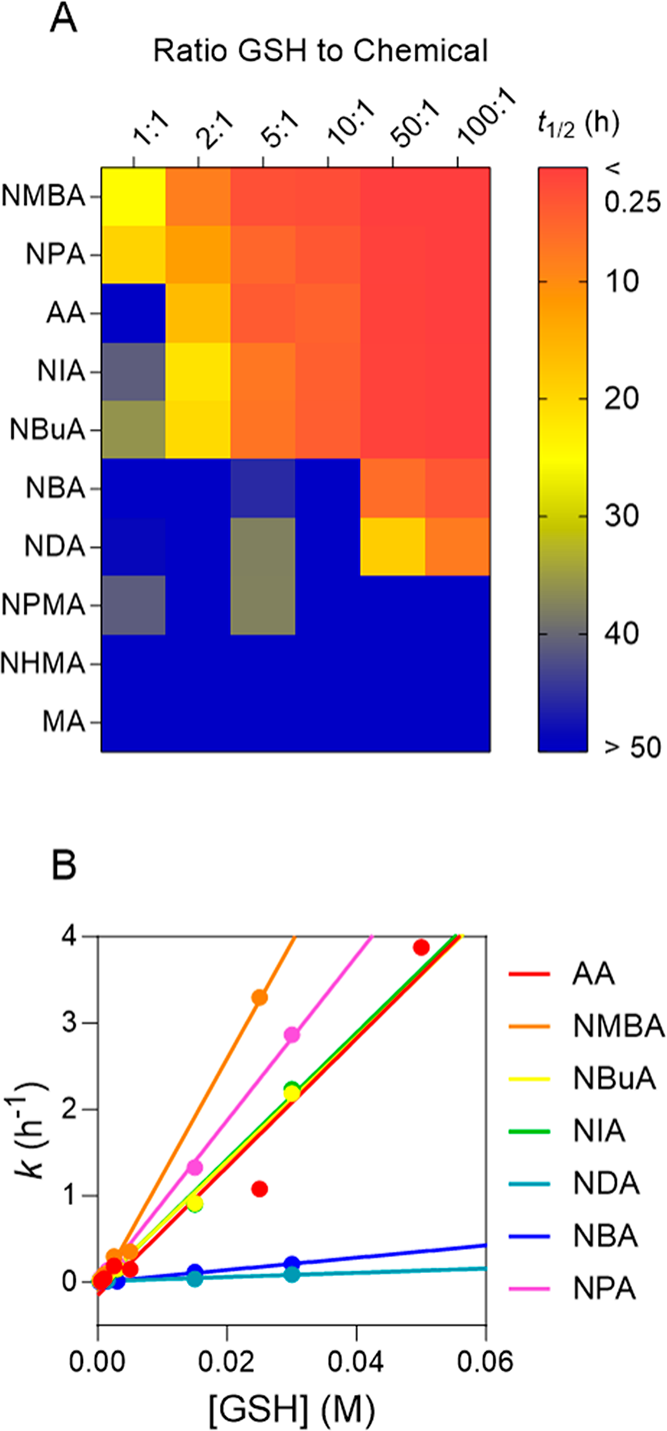
Degradation kinetics of the test chemicals with glutathione (GSH). (A) Degradation half-lives (*t*_1/2_) of the test chemicals with different concentrations of GSH. (B) Linear regression of pseudo-first-order degradation rate constants (*k*) plotted against the concentration of GSH.

Eight chemicals showed *t*_1/2_ below 50 h with the biological nucleophile glutathione (GSH) (Figures S9 and S10), and reactions were much faster than with 2DG. The associated degradation rate constants (*k*) are shown in Table S5. Figure 5A shows the degradation half-lives (*t*_1/2_) of the chemicals incubated with different concentrations of GSH. The concentration of the test chemical was kept constant so that only the ratio of nucleophile to chemical was changed. The lowest ratio of nucleophile to chemical was 1:1, and the highest was 100:1. MA and NHMA were not reactive and showed *t*_1/2_ above 50 h for all GSH concentrations, and NPMA showed *t*_1/2_ of 41.0 h (GSH/NPMA = 1:1) and 37.4 h (GSH/NPMA = 5:1), but *t*_1/2_ above 50 h for the other GSH ratios. For the other seven chemicals, *t*_1/2_ decreased with increasing concentration of GSH. NMBA showed the overall lowest *t*_1/2_ and NDA the highest *t*_1/2_ (Table S5). At the highest concentration of GSH, the reaction of NMBA was faster than the sample preparation time (15 min) so that *t*_1/2_ could not be determined (Table S5). The measured pseudo-first-order degradation rate constants (*k*) were plotted against the concentration of GSH for all chemicals that showed degradation (Figure 5B). Linear regression was used to derive the second-order degradation rate constants (*k*_GSH_) of the chemicals from the slope of this plot (eq 9).

The intercept of the fit gave the reaction constant of the reaction with water (*k*_H_2_O_). For all chemicals, the intercept was close to 0 (Table S5), so the reaction with water is negligible for all chemicals. This is consistent with the observation that none of the substances showed degradation in PBS (Figures S7–S10). NMBA showed the highest *k*_GSH_ (Table S5, 134.800 M^–1^ h^–1^) and NDA the lowest (Table S5, 2.574 M^–1^ h^–1^). As NBMA has two reactive groups, it had a *k*_GSH_ approximately twice as high as AA, NBuA, and NIA because, unlike the other test substances, it can react with two GSH molecules. NPMA, NHMA, and MA showed no degradation with GSH and no *k*_GSH_ values could be determined.

It is known that acrylamides react with GSH via a Michael addition.^17,18,69^ The resulting Michael adducts were identified as common metabolites of acrylamides, making the reaction with GSH an important detoxification process of acrylamides *in vivo*.^70,71^ Mass spectrometry was used to identify the transformation products of the reaction with GSH. As expected, Michael conjugation products with GSH were found for eight test chemicals. Chemical structures and MS/MS spectra of the conjugation products are shown in Figure S11. No conjugation products were found for MA and NHMA. Since NBMA has two reactive groups, only the conjugation product with two GSH molecules was found, and the conjugation product with one GSH molecule could not be detected for this chemical. The relative amount of GSH-conjugate was measured for two GSH/acrylamide ratios (5:1 and 100:1) and three time points (1, 4, and 24 h). Figure S12 shows that at a ratio GSH/acrylamide of 5:1, the relative amount of GSH-conjugate increased over time for GSH conjugates of AA, NMBA, NBuA, NIA, NDA, NBA, and NPA. At a GSH/acrylamide ratio of 100:1, relative amounts of GSH-conjugate were already high at the shortest incubation time and showed higher variation. For AA, NMBA, NBuA, NIA, and NPA, the relative amount of GSH-conjugate decreased slightly over time. The high excess of GSH accelerates the conjugation reaction, resulting in high relative amounts of GSH-conjugate in the 100:1 samples. The GSH conjugates appear to be further degraded over time, which is why their relative amount decreases, but the resulting transformation products could not be identified. GSH conjugates of NPMA could only be found at a GSH/acrylamide ratio of 100:1.

To rationalize the GSH reactivity of the chemicals, quantum chemical calculations were performed and the charge densities of selected atoms (*q*(C_α_), *q*(C_β_), and *q*(C_1_)) and the energy of the lowest unoccupied orbitals (ε_LUMO_) were calculated (Table S6, Figure S13). The methacrylamides (MA, NPMA, and NHMA), which showed no reactivity with GSH in the experiment, had calculated *q* for C_α_, which were significantly less negative than those for the acrylamides (factor 4). So, the electron-donating effect of the methyl group causes the reduced reactivity of these chemicals.^72,73^ For the differently substituted acrylamides, ε_LUMO_ and the *q* of the atoms C_α_, C_β_, and C_1_ showed no difference (Table S6). Nevertheless, NDA and NBA had much lower *k*_GSH_ values than the other acrylamides (factors 35 and 10, respectively). In the case of NDA, the formation of the intermediate state of the reaction with GSH is sterically hindered by the two ethyl groups on the nitrogen, which leads to a deceleration of the reaction rate.^74^ The low reactivity of NBA was surprising at first glance since its structure and the results of the quantum chemical calculations (Table S6) are very similar to those of the highly reactive NPA. These observations could be explained by looking at the depiction of the LUMO (Figure S13). While for NPA the orbitals of the phenyl ring and the α,β-unsaturated carbonyl group are clearly separated from each other, for NBA the orbitals of the phenyl ring merge with those of C_α_ and C_β_ lowering the electrophilicity, which explains the low reactivity.

### Comparison of Toxicity and Reactivity

**Figure 6:**
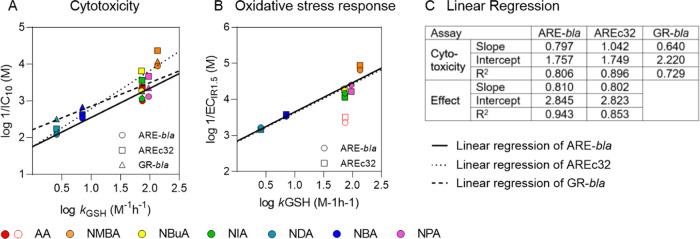
Linear regression of cytotoxicity (log 1/IC_10_) (A) and activation of the oxidative stress response (EC_IR1.5_) (B) plotted against reactivity with GSH (*k*_GSH_). (C) Regression parameters of linear regression. AA was excluded from the fit of the oxidative stress response. No *k*_GSH_ could be quantified for MA, NPMA, and NHMA.

All methacrylamides and NDA and NBA showed the lowest effects and acted as baseline toxicants in all assays (Table 1 and Figure 3). These chemicals also showed no reactivity (methacrylamides) or low reactivity to GSH (NDA and NBA, Table S5). For the acrylamides, the measured effect concentrations for cytotoxicity (log 1/IC_10_) and the activation of the oxidative stress response (log 1/EC_IR1.5_) increased log-linearly with an increase in log *k*_GSH_ (Figure 6). Methacrylamides are not included, as they did not show reactivity toward GSH.

The log-linear relationship of the cytotoxicity (Figure 6A) or oxidative stress response (Figure 6B) and the reactivity toward GSH was shown by linear regression (Figure 6C). AA was excluded from the fit of the oxidative stress response since it showed lower effects than predicted by the fit (Figure 6B). The reason for the deviation of AA from the fit is unclear, but degradation in the medium or loss due to volatilization over the time of the assay can be excluded (Figure 4). Thus, cellular processes such as metabolism must be responsible for the low effects. However, these processes must occur to the same extent in both cell lines since no difference in the effect concentrations was observed for the different assays. Further tests on acrylamide metabolism are necessary to explain this observation. Even though a test set of 7 chemicals (cytotoxicity) or 6 chemicals (oxidative stress response) is rather small for the establishment of a QSAR, the *R*^2^ of the linear regression were between 0.792 and 0.943 for the three cell lines (Figure 6C). While the fits for the activation of the oxidative stress response were almost identical in both cell lines, the fits for cytotoxicity differed slightly for the three cell lines tested, which can be explained by the generally higher variability of the cytotoxicity measurement. We therefore conclude that the predominant MOA of the test substances is the formation of ROS, which leads to an activation of the oxidative stress response. In addition, a decrease in the intracellular GSH level by direct reaction of GSH with the test substances disrupts the intracellular redox homeostasis and thus the protection against ROS. In this study, no intracellular GSH or ROS levels were measured; therefore, a confirmation of the proposed MOA in the tested cells is not possible. However, both *in vitro* and *in vivo* studies have shown that exposure to acrylamides leads to an increase in intracellular ROS levels and a decrease in GSH levels.^75−77^ For other reactive chemicals, another possible mechanism of activation of the Nrf-2/Keap-1 pathway has been described. For example, Dinkova-Kostova et al.^78^ and Suzuki et al.^37^ have shown that some electrophilic chemicals react directly with cysteine residues of Kelch-like ECH-associated protein 1 (Keap-1), an important protein of the oxidative stress signal chain and thus trigger the activation of the oxidative stress response.^79^ In this case, the reactivity of the chemicals with GSH can serve as a measure of the reactivity with cysteine-rich proteins in the cell.^80^ However, this mechanism has not been verified for acrylamide or related chemicals.^81,82^

## Conclusions

In this study, we investigated the cytotoxicity and activation of oxidative stress response via the Nrf-2/Keap-1 pathway of seven acrylamides and three methacrylamides and compared both *in vitro* effects with the reactivity toward the biological nucleophile GSH. The identification of the molecular initiating event is important for the interpretation of *in vitro* results of reactive chemicals with respect to possible adverse effects in humans. DNA damage and reactivity toward proteins or peptides are important MOAs of reactive chemicals. The softness or hardness of the electrophile determines the preferred reaction partner and thus the toxic effect.^24^ DNA damage is caused mainly by the reaction of hard electrophiles such as epoxides or organochlorides with DNA bases, whereas soft electrophiles such as the acrylamides tested in this study preferentially react with soft nucleophiles such as cysteine residues of cellular proteins or peptides.^25^ Reaction of the test chemicals with the hard nucleophile 2DG was much slower than the incubation time of a cell-based *in vitro* bioassay (24 h). Therefore, a direct genotoxic effect of the test chemicals through the formation of DNA adducts could be excluded. Nevertheless, acrylamide shows carcinogenic effects *in vivo*, because it can be metabolized to the hard nucleophile glycidamide, which shows reactivity toward DNA.^22,40^ For a complete evaluation of the carcinogenicity of the test chemicals, further genotoxicity tests, such as the micronucleus test or a reporter gene assay for the induction of the tumor suppression factor p53 after metabolic activation, would be required to assess the mutagenic potential of possible metabolites. In addition, external metabolization using, for example, S9 or microsomes would be necessary, as the reporter gene cell lines used show only low cytochrome P450 activity.^35,83^

For the acrylamides tested in this study, the activation of the oxidative stress response via the Nrf-2/Keap-1 pathway was probably triggered by the intracellular formation of ROS as well as a disturbance of the redox balance by the reduction of the intracellular GSH level.^77^ This adverse outcome pathway (AOP)^84^ has already been shown for different adverse effects, such as hepatotoxicity^85^ and neurotoxicity^76^ of acrylamides. However, for some chemicals, the oxidative stress response can also be triggered by direct binding of the test chemicals to Keap-1, which has been identified as a molecular initiating event for skin sensitization,^79^ but direct reactions with Keap-1 and acrylamides have not yet been demonstrated.^81^ The results of this study can be used to deduce the probable behavior of the chemicals in humans and their potential effects on human health. The relationship between reactivity and toxicity of electrophiles has been extensively studied in the past and a number of QSAR models are available for predicting toxicity in different *in vitro* systems.^31,86,87^ Comparable QSARs have also been developed to predict the toxicity of acrylates in *in vitro* cell lines.^88^ We have found a linear relationship between the reactivity of the chemicals toward GSH and the activation of oxidative stress response *in vitro* (Figure 6). This result can be used to predict *in vitro* effects for other test chemicals, although a larger and more diverse set of test chemicals would be needed for reliable quantitative prediction and an elucidation of the MOA of the test chemicals. Nevertheless, the measurement of *k*_GSH_, which can be done in HT format, together with quantum chemical calculations of the chemical reactivity allows a suitable estimation of the bioassay results and simplifies their interpretation.

## Supporting Information Available

Test chemicals, layout of the robot, analytical methods, concentration–response curves, uptake kinetics, pin-water distribution ratios, degradation kinetics, degradation rate constants, structures and MS/MS spectra of transformation products, quantum chemical calculations, and lowest unoccupied molecular orbitals (PDF)
