# Supplementary material for: Reactivity of Acrylamides Causes Cytotoxicity and Activates Oxidative Stress Response
Source: Chem Res Toxicol. 2023 Aug 2;36(8):1374–85. doi: 10.1021/acs.chemrestox.3c00115 (PMC10445285; doi:10.1021/acs.chemrestox.3c00115)
Supplement: Supplementary file 1 — tx3c00115_si_001.pdf [file tx3c00115_si_001.pdf]

Supporting Information

Reactivity of Acrylamides Causes Cytotoxicity and Activates Oxidative Stress Response

Julia Huchthausen[†], Beate I. Escher[†, §,*], Nico Grasse[¶], Maria König[†], Stephan Beil[‡], Luise Henneberger[†]

[†] Helmholtz Centre for Environmental Research – UFZ, Department of Cell Toxicology, Permoserstr. 15, 04318 Leipzig, Germany

[§] Eberhard Karls University Tübingen, Environmental Toxicology, Department of Geosciences, 72076 Tübingen, Germany

[¶] Helmholtz Centre for Environmental Research – UFZ, Department of Analytical Chemistry, Permoserstr. 15, 04318 Leipzig, Germany

[‡] Technische Universität Dresden, Institute of Water Chemistry, 01069 Dresden, Germany

*Corresponding author: Beate Escher (beate.escher@ufz.de)

Table of contents

Table S1: Test chemicals S3

Table S2: Labware, solvents and experimental parameters for solid-phase microextraction. S3

Figure S1: Scheme of the deck layout of the Hamilton Microlab Star robotic system. S4

Table S3: Liquid chromatography (LC) and mass spectrometry (MS) parameters of the test chemicals. S5

Figure S2: Concentration-response curves (CRC) of the ARE-*bla* bioassay. S6

Figure S3: Concentration-response curves (CRC) of the GR-*bla* bioassay. S9

Figure S4: Concentration-response curves (CRC) of the AREc32 bioassay. S12

Figure S5: Comparison of inhibitory and effect concentrations between the assays. S12

Text S1: SPME method validation. S13

Figure S6: Uptake kinetics of the test chemicals into the BioSPME pins. S14

Table S4: Logarithmic pin-water distribution ratios (log $D_{pin/w}$), recovery, extracted mass (m_{ex}) and time until 95 % equilibrium is reached ($t_{95\%}$) for all test chemicals. S15

Figure S7: Degradation kinetics of AA, NMBA and MA in 2′-deoxyguanosine (2DG) solutions in PBS and in PBS alone. S15

Figure S8: Degradation kinetics of NBuA, NIA, NDA, NBA, NPMA, NPA and NHMA in 2′-deoxyguanosine (2DG) solutions in PBS and in PBS alone. S16

Figure S9: Degradation kinetics of AA, NMBA and MA in glutathione (GSH) solutions in PBS and in PBS alone. S16

Figure S10: Degradation kinetics of NBuA, NIA, NDA, NBA, NPMA, NPA and NHMA in glutathione (GSH) solutions in PBS and in PBS alone. S17

Table S5: Experimental pseudo first-order degradation rate constants (k) at different 2′-deoxyguanosine (2DG) and glutathione (GSH) concentrations and second-order degradation rate constants of the reaction of test chemicals with GSH (k_{GSH}) and degradation rate constants of the reaction of the test chemicals with water (k_{H2O}) S18

Figure S11: Structure, ionization mode, retention time, confidence level and MS/MS spectra of identified glutathione conjugates of acrylamides. S19

Figure S12: Relative amount of glutathione conjugates of acrylamides plotted against the incubation time. S23

Table S6: Quantum chemical calculations of the test chemicals S24

Figure S13: Lowest unoccupied molecular orbitals (LUMO) of the test chemicals. S24

References S24

Table S1: Test chemicals* with ID, *CAS number, molar mass, provider, purity, logarithmic air-water partition constant (log K_{aw}), logarithmic liposome-water partition constant (log K_{lipw}) and desorption solvent for solid-phase microextraction (SPME) composed of acetonitrile (ACN) and MilliQ water.

**Chemical**	**ID**	**CAS**	**Molar mass [g/mol]**	**Provider**	**Purity [%]**	**Log K_{aw}, 37 °C [L/L]**	**Log K_{lipw} [a] [L/L]**	**Desorption solvent**
Acrylamide	AA	79-06-1	71.08	Sigma Aldrich	≤ 100	-4.52[a]	-0.22	10/90 ACN/MilliQ
N,*N'*-Methylene-bisacrylamide	NMBA	110-26-9	154.17	Sigma Aldrich	≤ 100	-9.69[a]	-0.56	10/90 ACN/MilliQ
N-(Butoxy-methyl)-acrylamide	NBuA	1852-16-0	157.21	Sigma Aldrich	≤ 100	-5.50[a]	1.06	10/90 ACN/MilliQ
N-(Isobutoxy-methyl)-acrylamide*	NIA	16669-59-3	157.21	Sigma Aldrich	≥ 70 - 90	-5.17[a]	1.32	10/90 ACN/MilliQ
				TCI	> 98			
N,*N*-Diethyl-acrylamide	NDA	2675-94-7	127.19	Sigma Aldrich	≤ 100	-3.73[a]	0.3	10/90 ACN/MilliQ
Methacrylamide	MA	79-39-0	85.11	Sigma Aldrich	≤ 100	-4.40[a]	0.29	10/90 ACN/MilliQ
N-Benzyl-acrylamide	NBA	13304-62-6	161.20	Alfa Aeser	96	-5.95[a]	1.76	50/50 ACN/MilliQ
N-Phenylmeth-acrylamide	NPMA	1611-83-2	161.20	TCI	> 98	-6.16[a]	2.08	50/50 ACN/MilliQ
N-Phenyl-acrylamide	NPA	2210-24-4	147.17	TCI	> 98	-6.28[a]	1.57	50/50 ACN/MilliQ
N-(4-Hydroxy-phenyl)-methacrylamide	NHMA	19243-95-9	177.20	TCI	> 98	-8.57[a]	2.18	50/50 ACN/MilliQ

[*] Two different purity grades of N-(Isobutoxymethyl)acrylamide were used for the *in vitro* bioassays. For the reactivity tests, only the chemical from Sigma-Aldrich was used. [a] Partition constants were calculated using the UFZ-LSER database.[1]

Table S2: Labware, solvents and experimental parameters for solid-phase microextraction.

**Plate**	**Name**	**Labware**	**Solution**	**Experimental parameters**
0. Pin Device	Park Position	Deep-well reservoir with spacer and pin device	Empty	
1. Plate	Conditioning 1	Deep-well reservoir with spacer and lid	Isopropanol	20 min
2. Plate	Conditioning 2	Deep-well reservoir with spacer	MilliQ water	10 sec
3. Plate	Extraction	Deep-well plate with lid	Sample solutions	15 min, 1000 rpm, 37 °C
4. Plate	Wash	Deep-well plate	Empty or MilliQ water	5 sec
5. Plate	Desorption	Deep-well plate with lid	Desorption solvents	15 min, 1000 rpm, RT

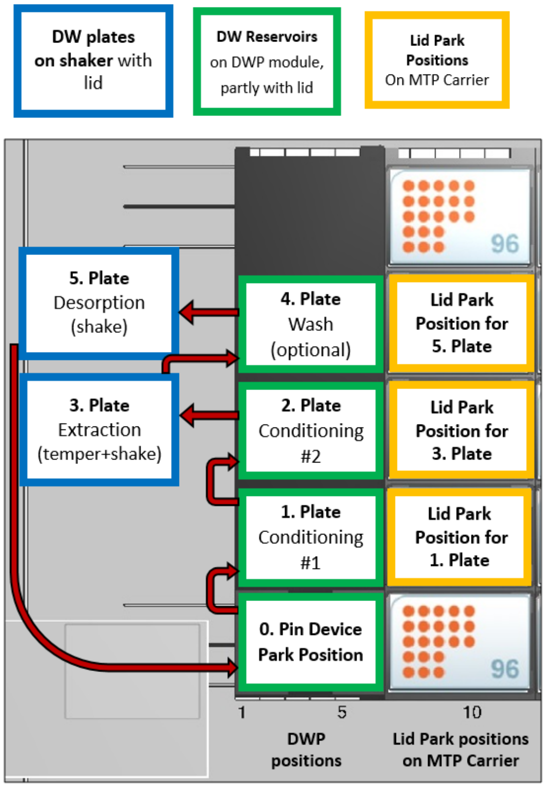

Figure S1: Scheme of the deck layout of the Hamilton Microlab Star robotic system. DW = deep-well, DWP = deep-well plate, MTP = multi-tier plate.

Table S3: Liquid chromatography (LC) and mass spectrometry (MS) parameters of the test chemicals. The injection volume was 1 µL for all chemicals. The eluents used were 5 % acetonitrile and 95 % MilliQ water with 0.1 % formic acid (C) and 95 % acetonitrile with 5 % MilliQ water with 0.1 % formic acid (D). An electrospray ion source (ESI) was used in positive mode.

Chemical	Eluent	Retention time [min]	Fragmentor voltage [V]	MRM transition (Collision energy [V])	Qualifier Ratio	Limit of quantification (LOQ) [ng/L]	Source conditions (Gas Temperature [°C]; Gas Flow [L/min]; Nebulizer [psi]; Capillary [V])
Acrylamide	98% C 2% D	0.47 ± 0.01	20	72.1 → 55.2/ 44.2 (10/14)	4.18 ± 0.04	1000	350; 13; 60; 1500
N,N'-Methylene-bisacrylamide	95% C 5% D	0.66 ± 0.01	20	155.1 → 72.1/ 55.1 (6/ 22)	64.88 ± 0.70	5000	350; 13; 40; 1500
N-(Butoxy-methyl)-acrylamide	70% C 30% D	0.76 ± 0.01	20	158.1 → 84.1/ 55.2 (2/ 18)	86.27 ± 3.87	1000	270; 7; 60; 1500
N-(Isobutoxy-methyl)-acrylamide	70% C 30% D	0.76 ± 0.01	20	158.1 → 84.1/ 55.2 (2/14)	86.35 ± 3.79	1000	270; 7; 60; 1500
N,N-Diethyl-acrylamide	70% C 30% D	0.65 ± 0.01	86	128.1 → 74.2/ 55.2 (10/18)	93.33 ± 1.89	5000	270; 7; 60; 1500
Methacrylamide	95% C 5% D	0.58 ± 0.01	20	86.1 → 58.2/ 41.2 (10/18)	46.24 ± 0.28	1000	350; 13; 60; 2000
N-Benzyl-acrylamide	75% C 25% D	0.75 ± 0.02	86	162.1 → 91.1/ 65.1 (18/40)	31.02 ± 0.51	1000	350; 13; 60; 1500
N-Phenylmeth-acrylamide	60% C 40% D	0.68 ± 0.01	86	162.1 → 134.1/ 69.1 (14/18)	87.26 ± 4.14	10000	230; 5; 60; 2500
N-Phenyl-acrylamide	70% C 30% D	0.72 ± 0.01	86	140.1 → 94.1/ 55.2 (18/22)	69.67 ± 2.38	5000	350; 7; 60; 1500
N-(4-Hydroxy-phenyl)-methacrylamide	85% C 15% D	0.80 ± 0.02	86	178.1 → 150.0/ 69.1 (14/18)	62.23 ± 3.42	5000	350; 13; 40; 4000

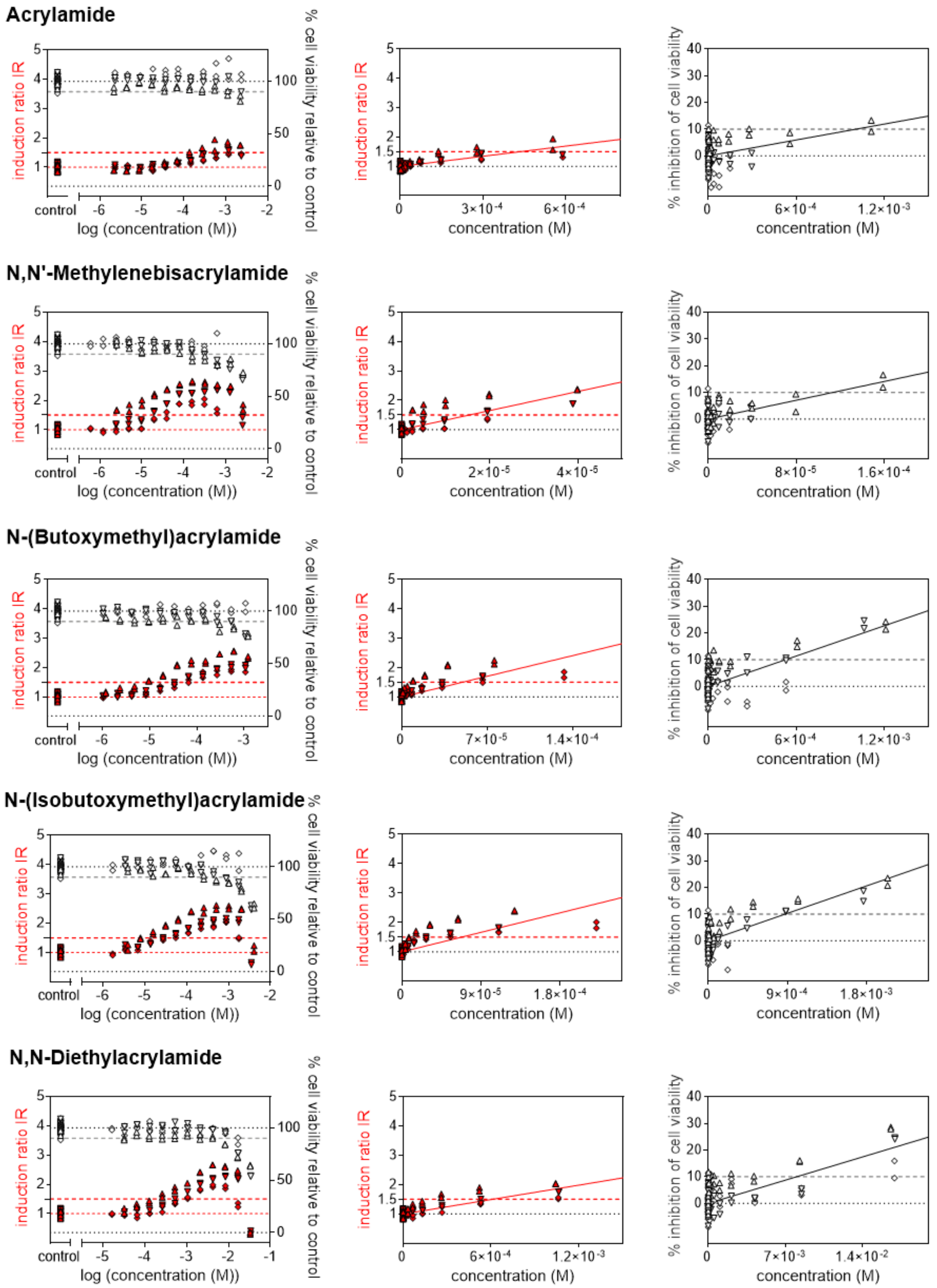

Figure S2 continued

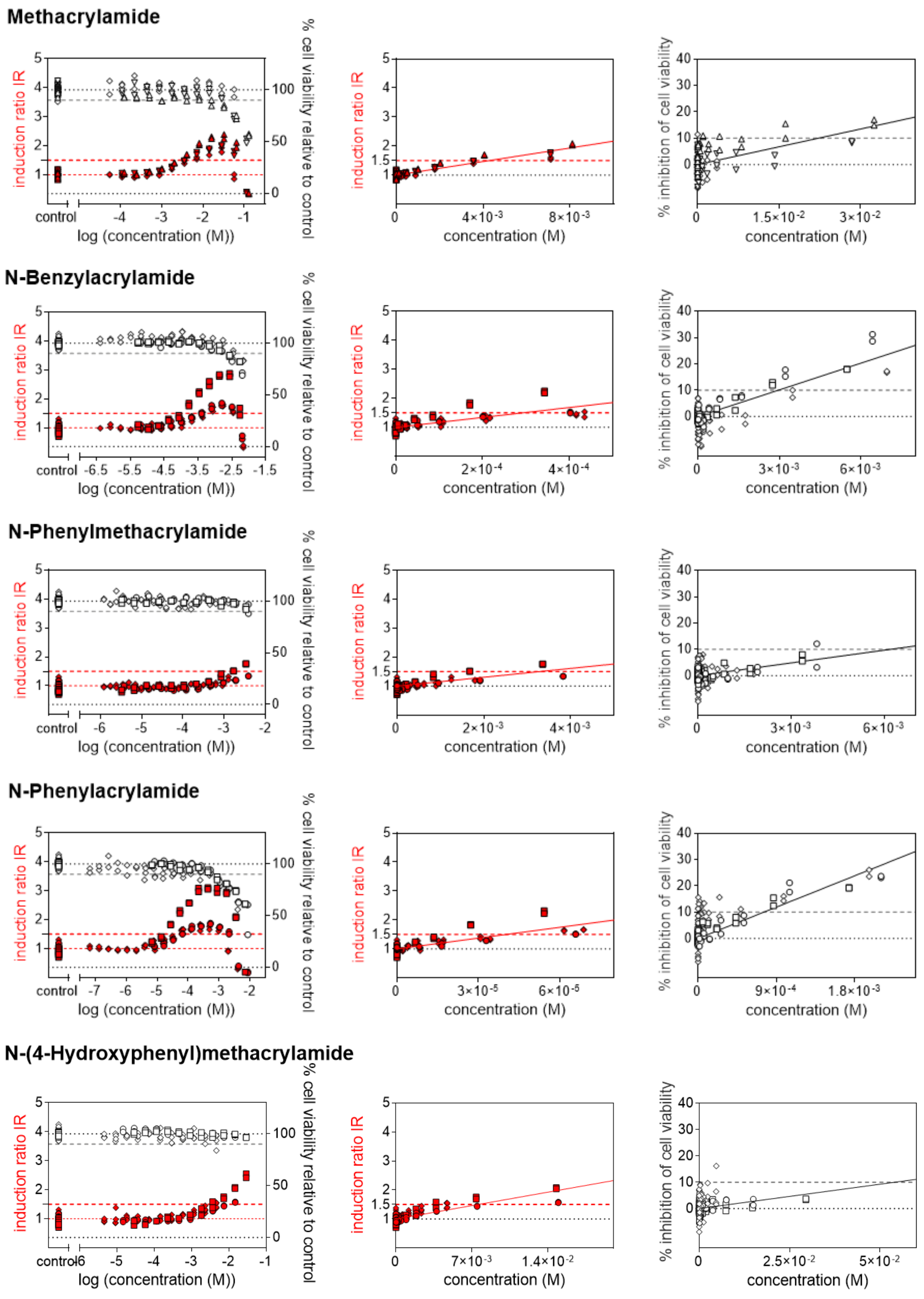

Figure S2 continued

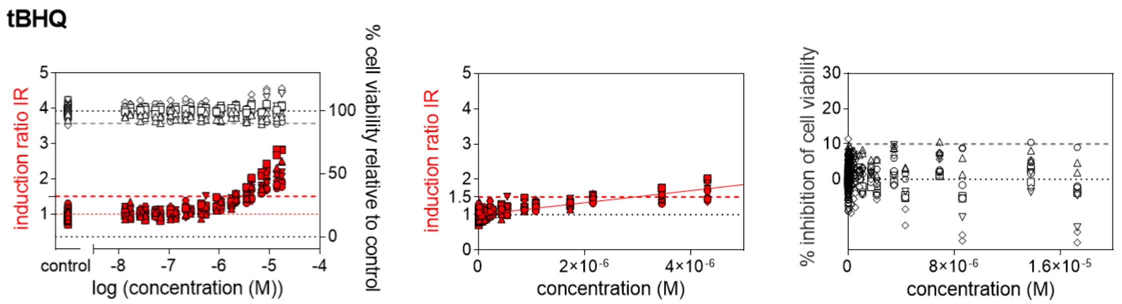

Figure S2: Concentration-response curves (CRC) of the ARE-bla bioassay. Left: Full CRC with induction ratio (red) and cell viability relative to control (white) plotted against the logarithm of the chemical concentration. Middle: Linear part of CRC with induction ratio plotted against chemical concentration. Right: Linear part of CRC with inhibition of cell viability plotted against chemical concentration. The different symbol shapes indicate different replicates of the experiment. Reference chemical tert-butylhydroquinone (tBHQ).

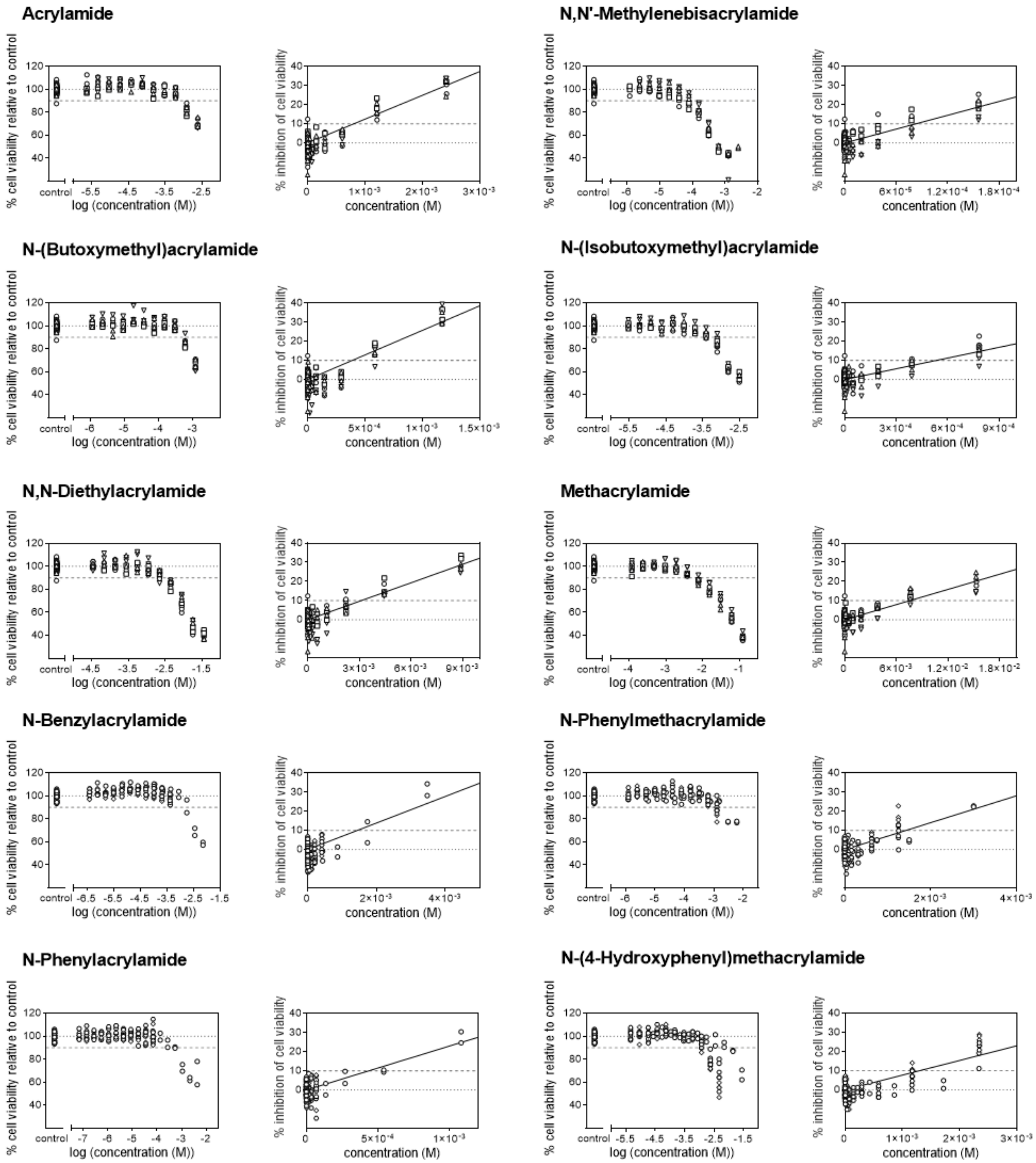

Figure S3: Concentration-response curves (CRC) of the GR-bla bioassay. Left: Full CRC of cell viability relative to control plotted against the logarithm of the chemical concentration. Right: Linear part of CRC with inhibition of cell viability plotted against chemical concentration. The different symbol shapes indicate different replicates of the experiment. Activation of the glucocorticoid receptor (GR) was not monitored.

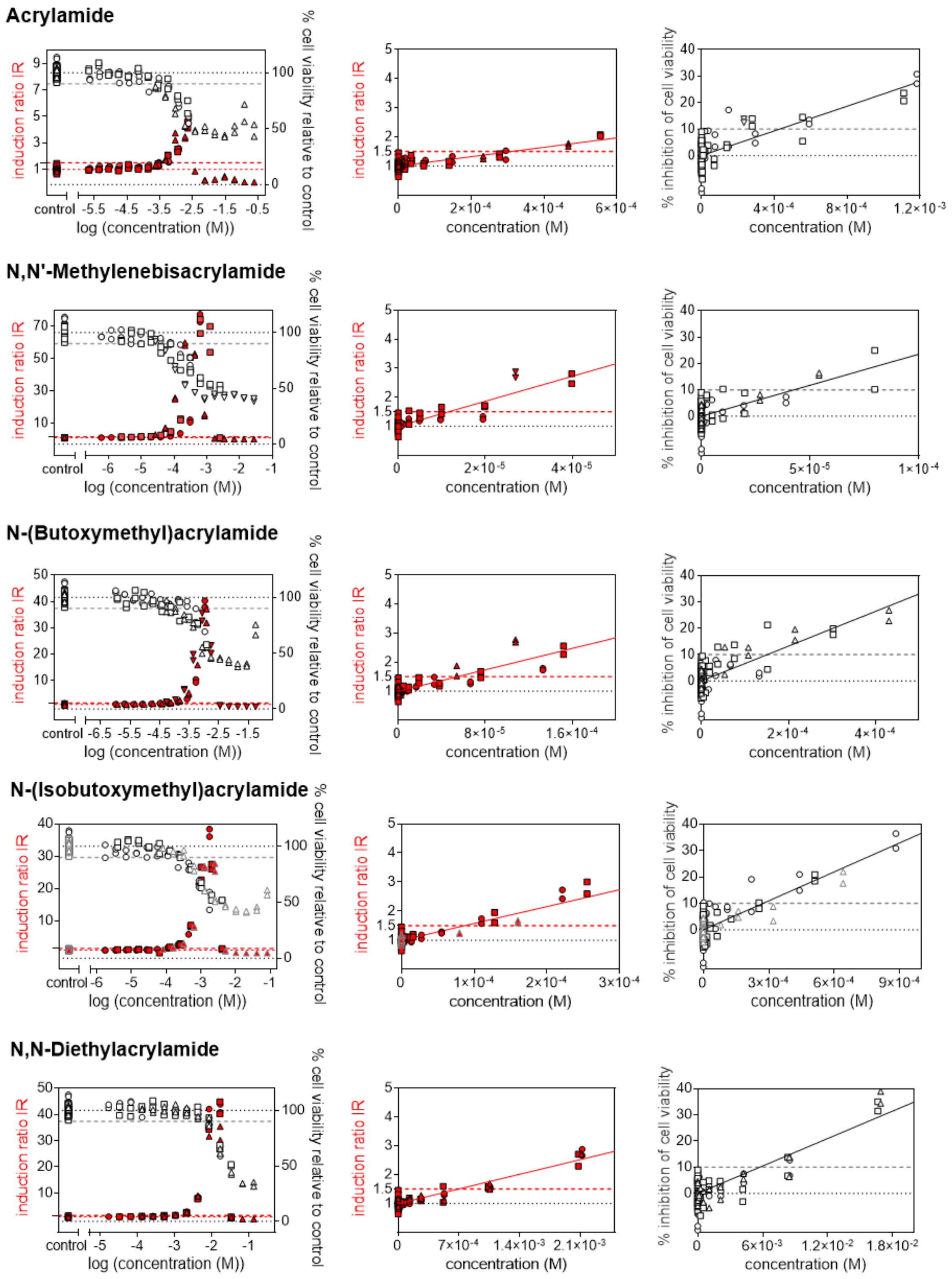

Figure S4 continued

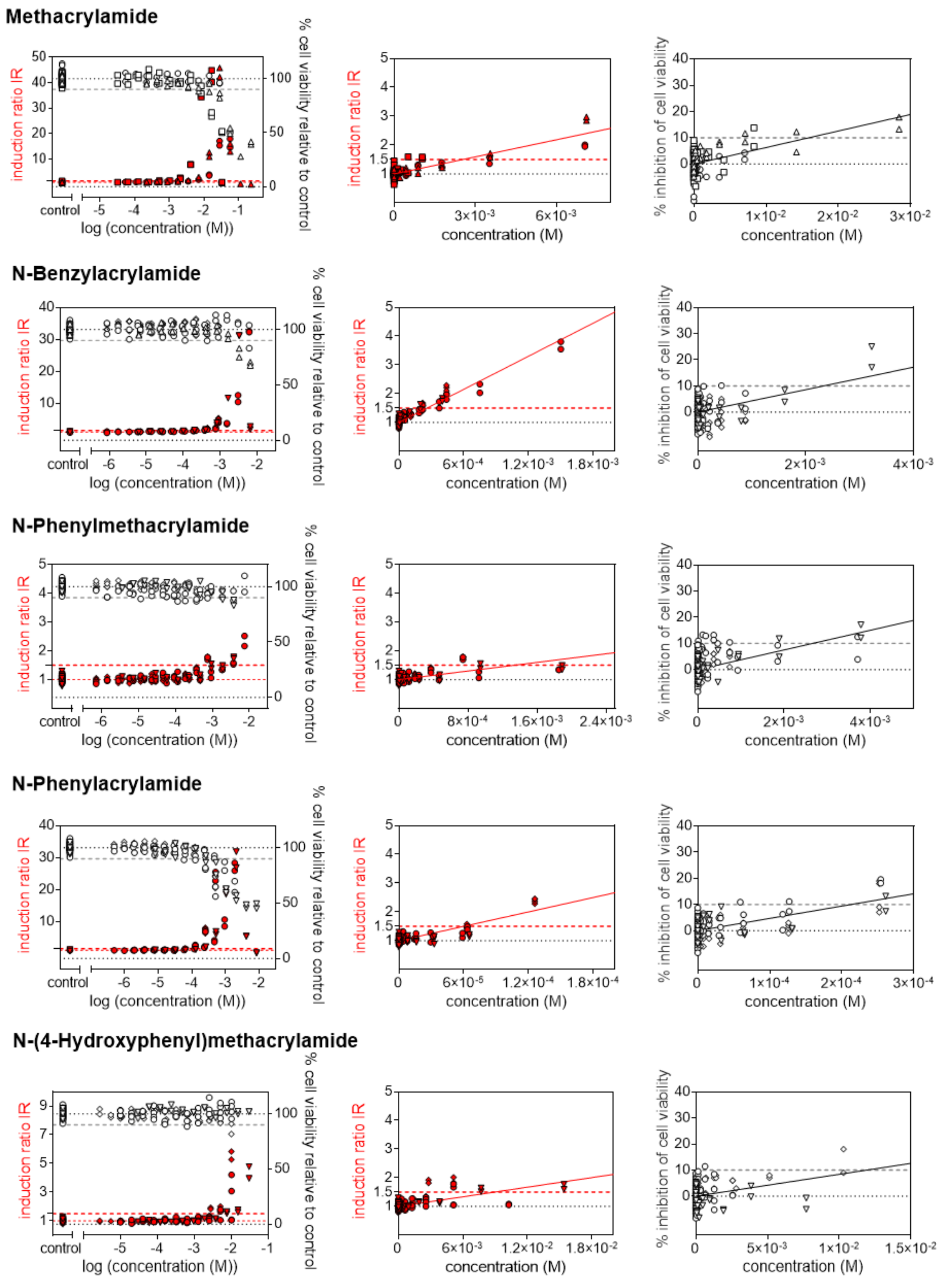

Figure S4 continued

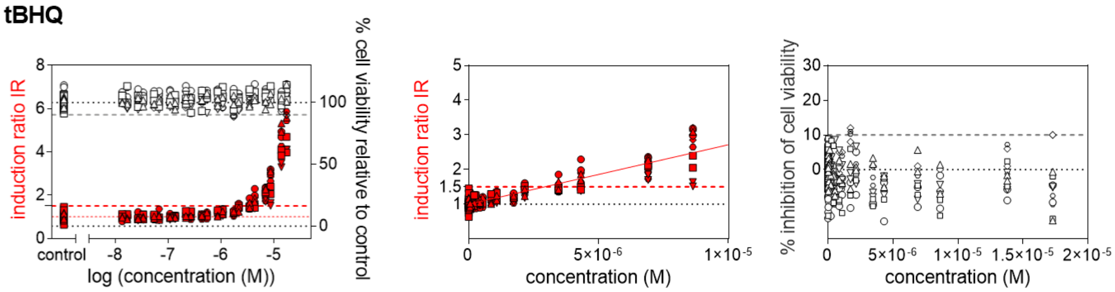

Figure S4: Concentration-response curves (CRC) of the AREc32 bioassay. Left: Full CRC with induction ratio (red) and cell viability relative to control (white) plotted against the logarithm of the chemical concentration. Middle: Linear part of CRC with induction ratio plotted against chemical concentration. Right: Linear part of CRC with inhibition of cell viability plotted against chemical concentration. The different symbol shapes indicate different replicates of the experiment. The two purities of N-(isobutoxymethyl)acrylamide used (Table S1) are indicated by the by the symbols outlined in gray and black, respectively. Reference chemical tert-butylhydroquinone (tBHQ).

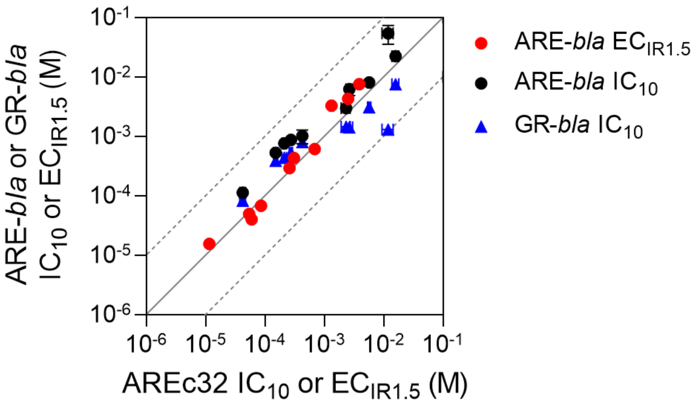

Figure S5: Comparison of inhibitory and effect concentrations between the assays. IC_{10} of ARE-bla (black) and GR-bla (blue) assays and $EC_{IR1.5}$ (red) of ARE-bla assay were plotted against IC_{10} and $EC_{IR1.5}$ of AREc32. The gray line shows a perfect agreement of the effect concentrations, the dashed gray lines indicate a deviation of a factor of ten.

Text S1: SPME method validation.

9 mL PBS were spiked with the chemical stock solutions to reach final concentrations of 5.0 $\times 10^{-4}$ M (AA, NMBA, MA) or 3.0 $\times 10^{-4}$ M (NBuA, NIA, NDA, NBA, NPMA, NPA, NHMA). Three replicates of 600 µL of each solution were pipetted into a glass-coated 96-deep well plate. The plate was preheated for 15 min at 37 °C and afterwards the chemicals were extracted using solid-phase microextraction (SPME). The same SPME method was used as for the other samples, but the experiment was repeated with varying extraction times (5, 15, 30, 45 min) while the desorption time was kept constant. PBS samples of all chemicals were diluted 1:10 with the respective desorption solvent. All sample and desorption plates were stored at 4 °C until instrumental analysis. Three aliquots of each PBS solution were diluted and injected into the LC without SPME extraction.

To determine the uptake kinetics of the C18 pins, the amount of chemical in the C18-coating of the pin (n_{pin}) was plotted against the extraction time (t). The data was fitted using equation S1 and $n_{\mathrm{pin}}(\mathrm{eq})$ is the amount of chemical in the pin coating at equilibrium and k_1 is the pin uptake rate constant.[2]

$$n_{\mathrm{pin}}(t) = n_{\mathrm{pin}}(\mathrm{eq}) \times (1 - \mathrm{e}^{-k_1 \times t}) \qquad \text{(S1)}$$

The time when 95 % equilibrium is reached ($t_{95\%}$) was calculated from k_1 using eq. S2.

$$t_{95\%} = \frac{\ln 0.05}{-k_1} \qquad \text{(S2)}$$

The pin-water distribution ratio ($D_{\mathrm{pin/w}}$) was calculated from the concentration in the pin coating (C_{pin}) and the concentration in the water phase (C_{w}) using eq. S3.

$$D_{\mathrm{pin}/\mathrm{w}} \left[\mathrm{L_w}/\mathrm{L_{pin}}\right] = \frac{C_{\mathrm{pin}}}{C_{\mathrm{w}}} \qquad \text{(S3)}$$

C_{des} is the concentration in the desorption solvent after desorption and V_{des} and V_{pin} are the volumes of desorption solvent and pin coating, respectively. V_{pin} was estimated from the pin dimensions and is approx. 80 nL. The extracted mass (m_{ex}) was calculated from C_{des} and V_{des} using eq. S4.

$$m_{\mathrm{ex}} = C_{\mathrm{des}} \times V_{\mathrm{des}} \qquad \text{(S4)}$$

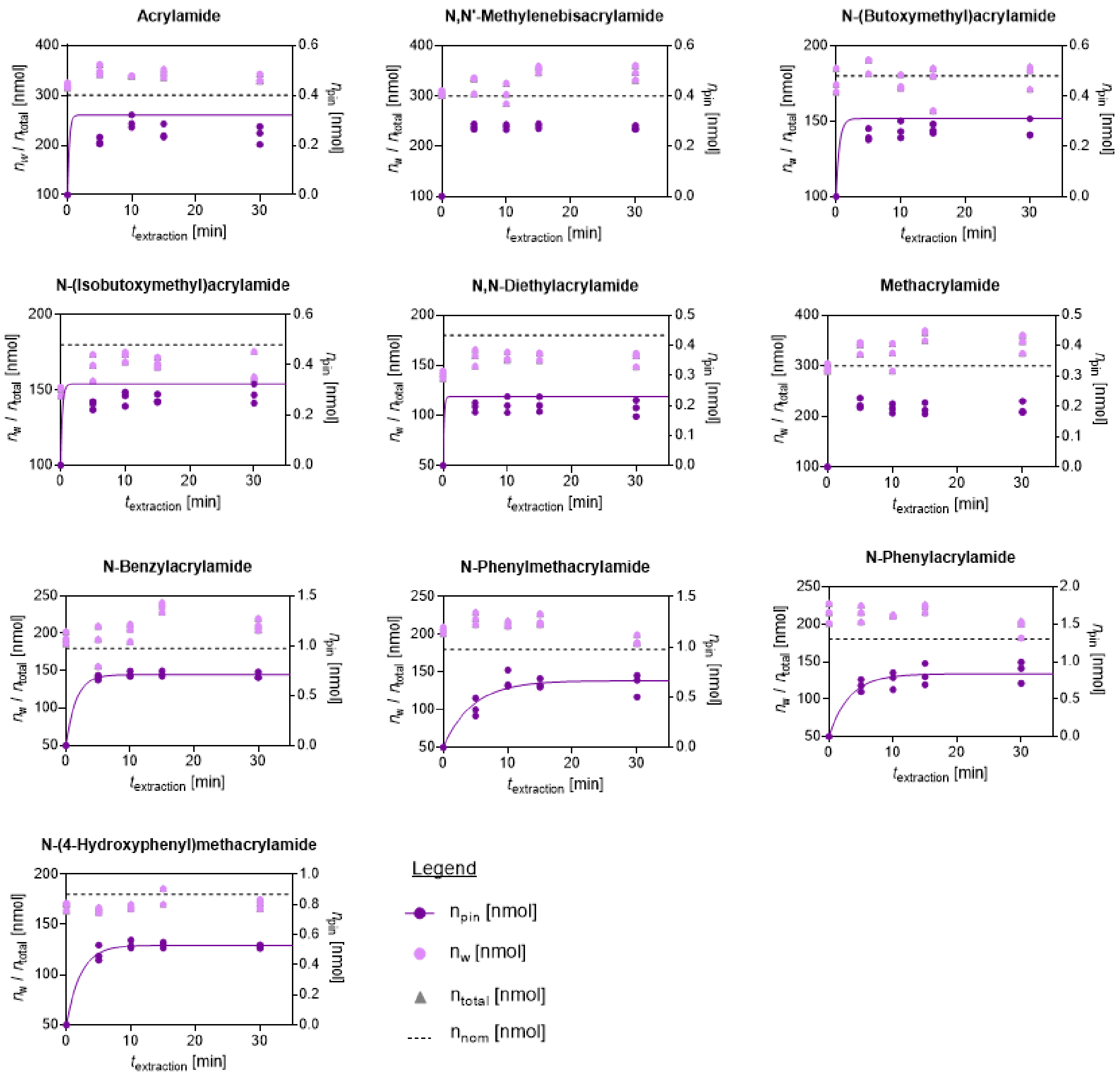

Figure S6: Uptake kinetics of the test chemicals into the BioSPME. The amount of chemical in the C18 pin coating (n_{pin}), the amount of chemical in the water (n_w) and the total amount of chemical (n_{total}) are plotted against the extraction time ($t_{extraction}$). The amount of chemical added to the sample solution (n_{nom}) is indicated by the dashed line.

Table S4: Logarithmic pin-water distribution ratios (log $D_{pin/w}$), recovery, extracted mass (m_{ex}) and time until 95 % equilibrium is reached ($t_{95\%}$) for all test chemicals. The log $D_{pin/w}$ and m_{ex} values are the average of the two higher time points (15 and 30 min) of the pin-water partitioning experiment (n= 6) and for the recovery values all time points were used.

**Chemical**	**log $D_{pin/w}$ [L_w/L_{pin}]**	**CV [%]**	**Recovery [%]**	**CV [%]**	**m_{ex} [ng]**	**CV [%]**	**$t_{95\%}$ [min]**
Acrylamide	0.73	6.63	112.43	3.71	17.54	12.15	≤ 5.00
N,N'-Methylene-bisacrylamide	0.77	2.85	109.02	7.41	42.61	3.16	≤ 5.00
N-(Butoxymethyl)-acrylamide	1.05	3.35	99.34	5.12	42.00	9.89	≤ 5.00
N-(Isobutoxymethyl)-acrylamide	1.09	4.78	91.09	5.97	42.99	10.64	≤ 5.00
N,N-Diethyl-acrylamide	0.97	4.38	86.06	5.63	25.02	12.30	≤ 5.00
Methacrylamide	0.61	9.17	110.56	8.16	16.35	9.14	≤ 5.00
N-Benzylacrylamide	1.38	2.09	114.41	10.50	114.45	3.97	≤ 5.00
N-Phenylmeth-acrylamide	1.36	5.24	116.93	5.71	101.82	12.14	13.55
N-Phenylacrylamide	1.48	4.63	117.85	5.80	124.77	15.66	9.64
N-(4-Hydroxyphenyl)-methacrylamide	1.35	0.93	94.80	4.17	93.19	3.01	6.61

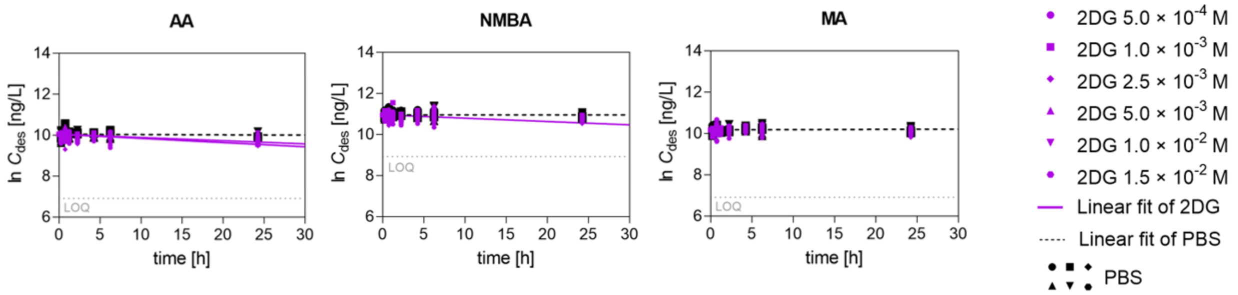

Figure S7: Degradation kinetics of AA, NMBA and MA in 2'-deoxyguanosine (2DG) solutions in PBS and in PBS alone. The natural logarithm of the chemical concentration was plotted against the incubation time. The violet colored symbols indicate the degradation with 2DG and the black symbols indicate the degradation in PBS. The linear regression of the data points is indicated by the violet lines (2DG) or by the black dotted lines (PBS). The different symbol shapes are different concentrations of 2DG. The gray dashed lines indicate the limit of quantification (LOQ).

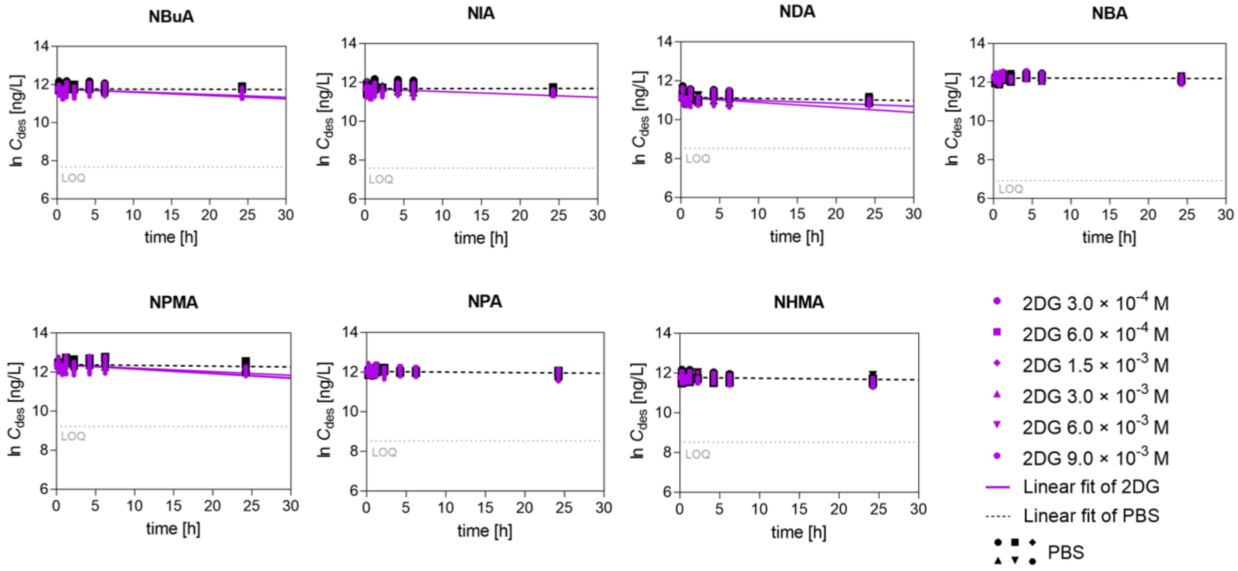

Figure S8: Degradation kinetics of NBuA, NIA, NDA, NBA, NPMA, NPA and NHMA in 2′-deoxyguanosine (2DG) solutions in PBS and in PBS alone. The natural logarithm of the chemical concentration was plotted against the incubation time. The violet colored symbols indicate the degradation with 2DG and the black symbols indicate the degradation in PBS. The linear regression of the data points is indicated by the violet lines (2DG) or by the black dotted lines (PBS). The different symbol shapes are different concentrations of 2DG. The gray dashed lines indicate the limit of quantification (LOQ).

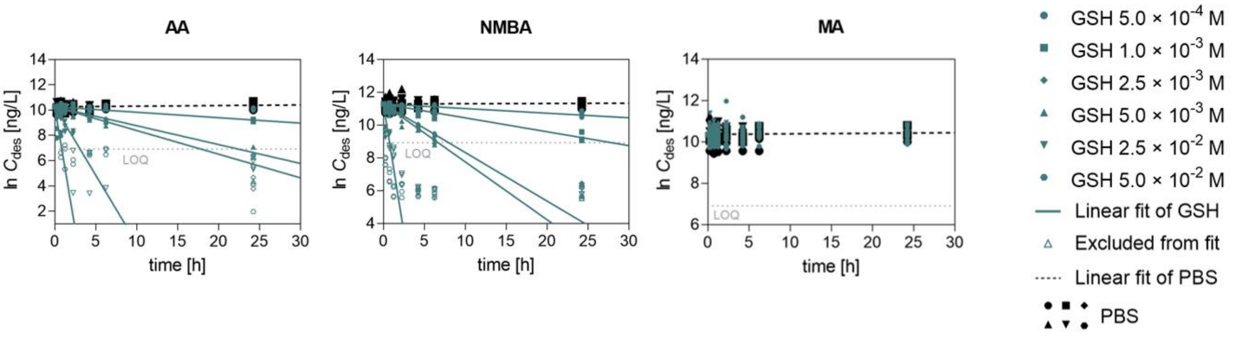

Figure S9: Degradation kinetics of AA, NMBA and MA in glutathione (GSH) solutions in PBS and in PBS alone. The natural logarithm of the chemical concentration was plotted against the incubation time. The turquoise-colored symbols indicate the degradation with GSH and the black symbols indicate the degradation in PBS. The linear regression of the data points is indicated by the turquoise lines (GSH) or by the black dotted lines (PBS). The different symbol shapes are different concentrations of GSH. The gray dashed lines indicate the limit of quantification (LOQ). If there was degradation, only data points above the LOQ were used for the linear regression.

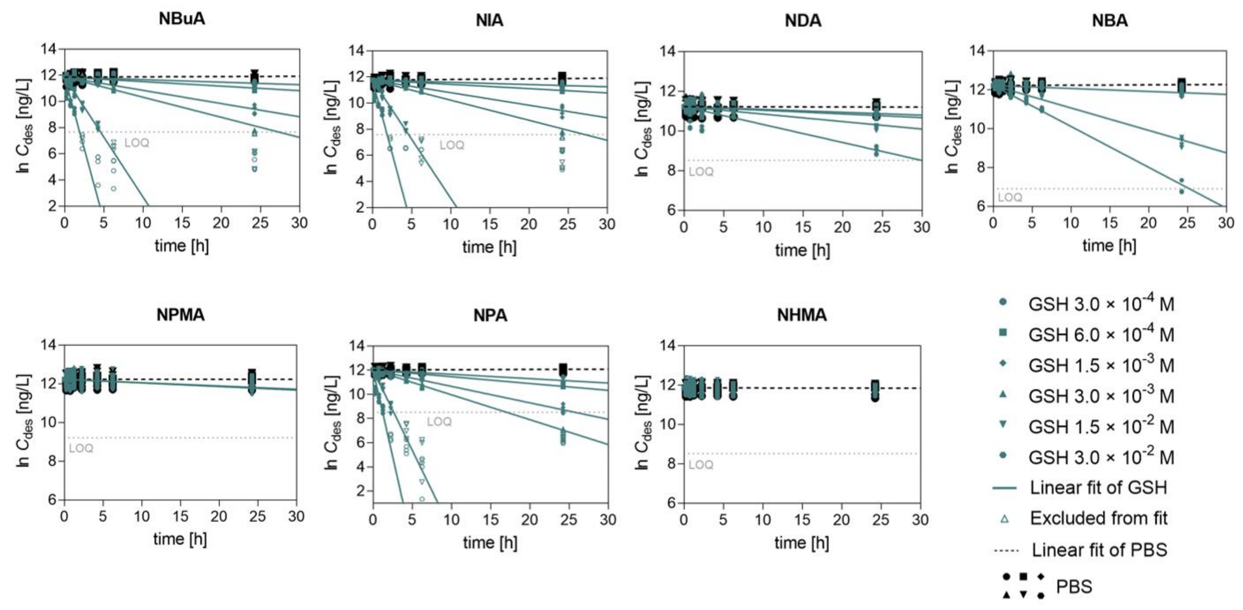

Figure S10: Degradation kinetics of NBuA, NIA, NDA, NBA, NPMA, NPA and NHMA in glutathione (GSH) solutions in PBS and in PBS alone. The natural logarithm of the chemical concentration was plotted against the incubation time. The turquoise-colored symbols indicate the degradation with GSH and the black symbols indicate the degradation in PBS. The linear regression of the data points is indicated by the turquoise lines (GSH) or by the black dotted lines (PBS). The different symbol shapes are different concentrations of GSH. The gray dashed lines indicate the limit of quantification (LOQ). If there was degradation, only data points above the LOQ were used for the linear regression.

Table S5: Experimental pseudo first-order degradation rate constants (k) at different 2′-deoxyguanosine (2DG) and glutathione (GSH) concentrations and second-order degradation rate constants of the reaction of test chemicals with GSH (k_{GSH}) and degradation rate constants of the reaction of the test chemicals with water (k_{H2O}) determined with eq. 9. No deg. = no degradation, n.d. = not determined.

Chemical	Ratio 2DG to chemical						k_{2DG} ($M^{-1}h^{-1}$)	k_{H2O} (h^{-1})
	1:1	2:1	5:1	10:1	20:1	30:1		
	k (h^{-1})	k (h^{-1})	k (h^{-1})	k (h^{-1})	k (h^{-1})	k (h^{-1})		
AA	0.010	0.016	0.020	0.005	0.006	0.011	n.d.	n.d.
NMBA	0.002	0.004	0.012	0.005	0.011	0.016	n.d.	n.d.
NBuA	0.006	0.006	0.002	0.010	0.015	0.017	n.d.	n.d.
NIA	0.007	0.011	0.005	0.011	0.015	0.013	n.d.	n.d.
NDA	0.002	0.007	0.015	0.005	0.013	0.026	n.d.	n.d.
MA	no deg.	0.000	0.010	no deg.	0.001	0.008	n.d.	n.d.
NBA	0.003	0.004	0.008	no deg.	0.002	0.005	n.d.	n.d.
NPMA	0.007	0.014	0.018	0.010	0.023	0.024	n.d.	n.d.
NPA	0.006	no deg.	0.004	no deg.	0.003	0.005	n.d.	n.d.
NHMA	0.010	0.013	0.009	0.009	0.007	0.009	n.d.	n.d.
Chemical	Ratio GSH to chemical						k_{GSH} ($M^{-1}h^{-1}$)	k_{H2O} (h^{-1})
	1:1	2:1	5:1	10:1	50:1	100:1		
	k (h^{-1})	k (h^{-1})	k (h^{-1})	k (h^{-1})	k (h^{-1})	k (h^{-1})		
AA	0.008	0.043	0.187	0.149	1.082	3.877	74.340	-0.150
NMBA	0.028	0.085	0.296	0.353	3.298	*	134.800	-0.104
NBuA	0.019	0.034	0.101	0.154	0.916	2.188	71.990	-0.036
NIA	0.017	0.033	0.096	0.154	0.904	2.230	73.250	-0.043
NDA	0.014	0.009	0.018	0.001	0.038	0.090	2.574	0.009
MA	0.000	no deg.	0.003	no deg.	no deg.	0.008	n.d.	n.d.
NBA	0.003	0.006	0.015	0.011	0.115	0.210	7.094	0.001
NPMA	0.017	0.007	0.019	no deg.	no deg.	0.007	n.d.	n.d.
NPA	0.036	0.056	0.136	0.206	1.329	2.866	95.180	-0.028
NHMA	0.013	0.006	0.006	no deg.	no deg.	no deg.	n.d.	n.d.

* No k could be determined, because the reaction was faster than the sample preparation time.

A: AA-GSH

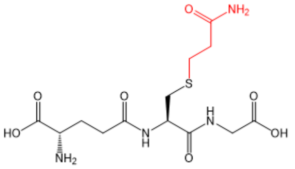

Ionization mode: ESI-

Retention time: 1.16 min

Confidence level: 3

$C_{13}H_{21}N_4O_7S$

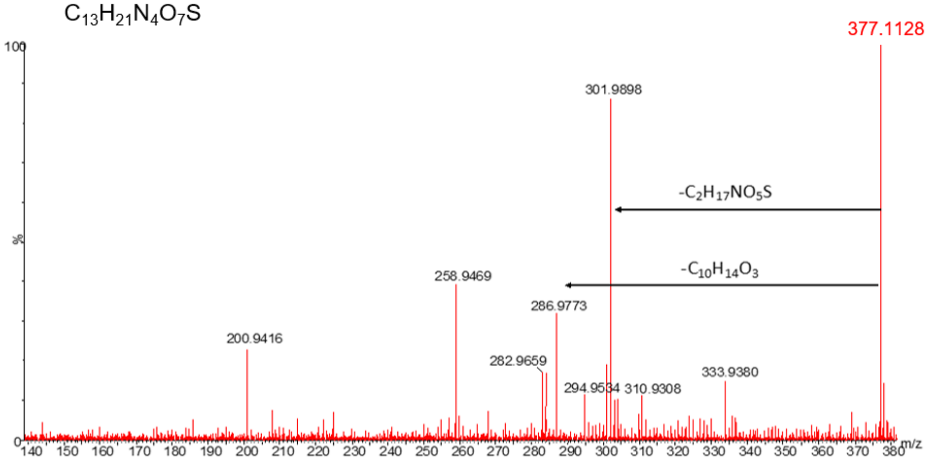

B: GSH-NMBA-GSH

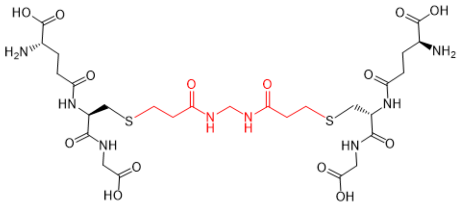

Ionization mode: ESI-

Retention time: 2.65 min

Confidence level: 3

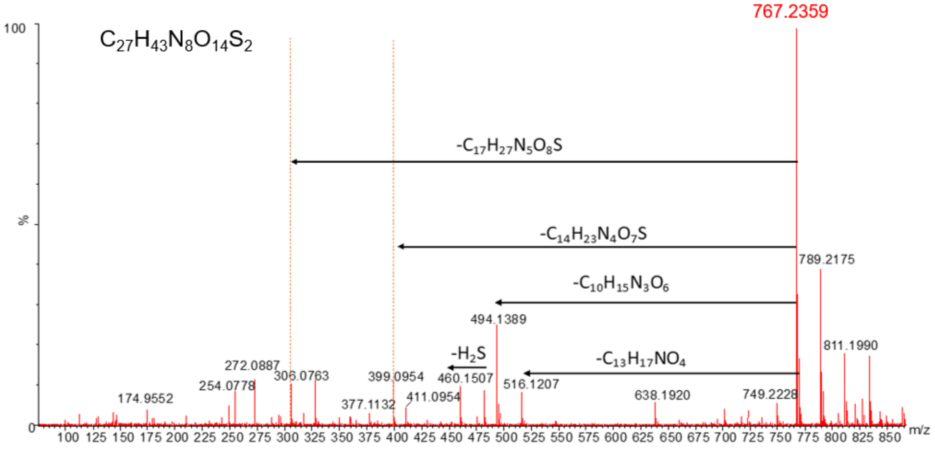

Figure S11 continued

C: NBuA-GSH

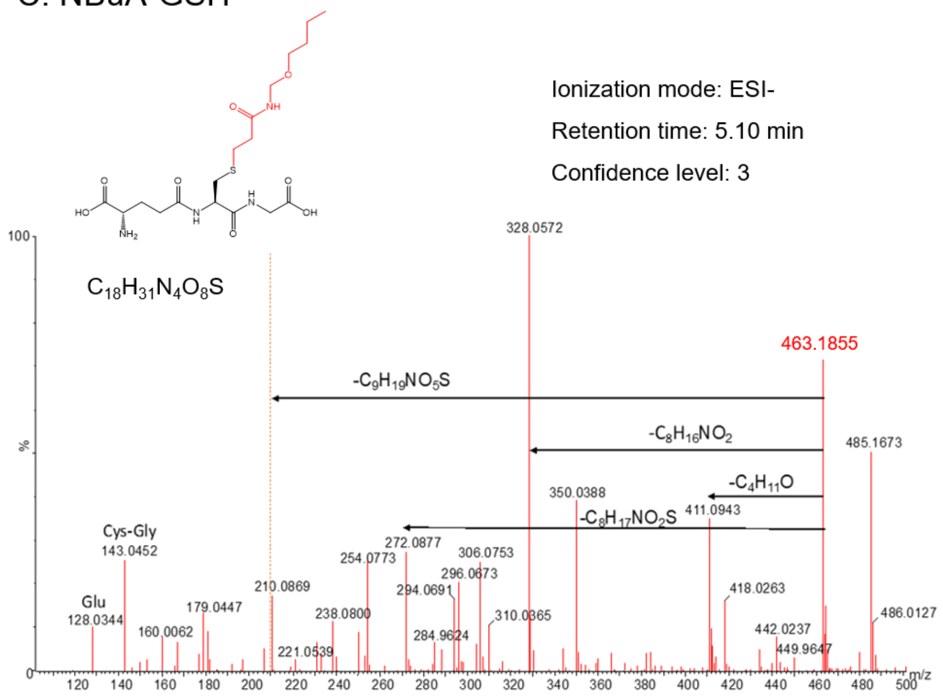

D: NIA-GSH

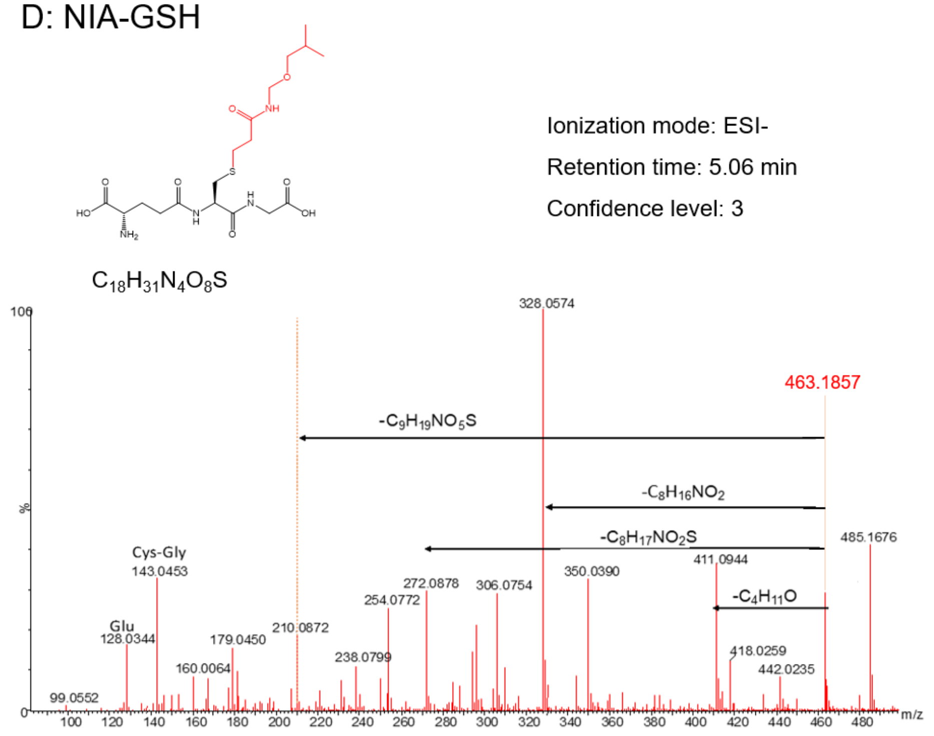

Figure S11 continued

E: NDA-GSH

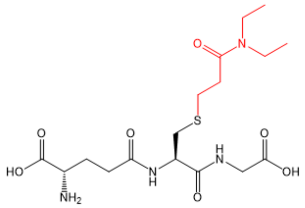

Ionization mode: ESI-

Retention time: 4.72 min

Confidence level: 3

$C_{17}H_{29}N_4O_7S$

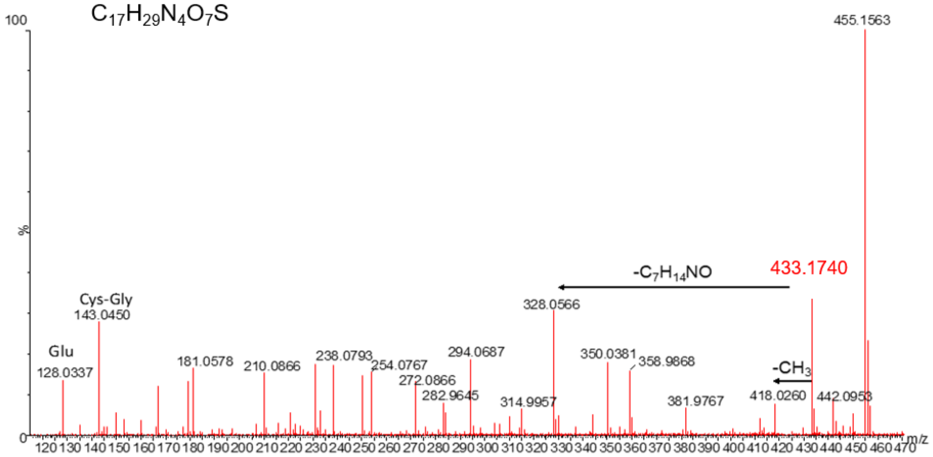

F: NBA-GSH

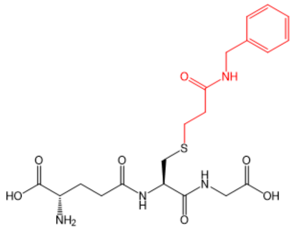

Ionization mode: ESI-

Retention time: 3.61 min

Confidence level: 3

$C_{20}H_{27}N_4O_7S$

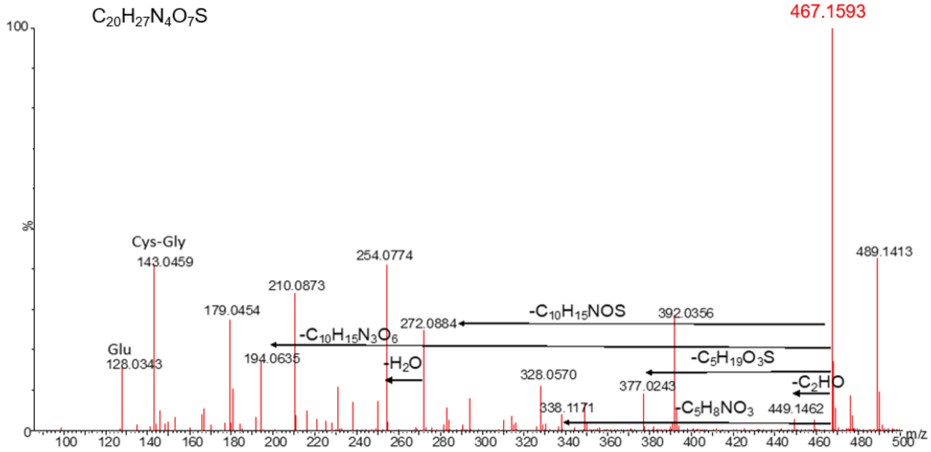

Figure S11 continued

G: NPMA-GSH

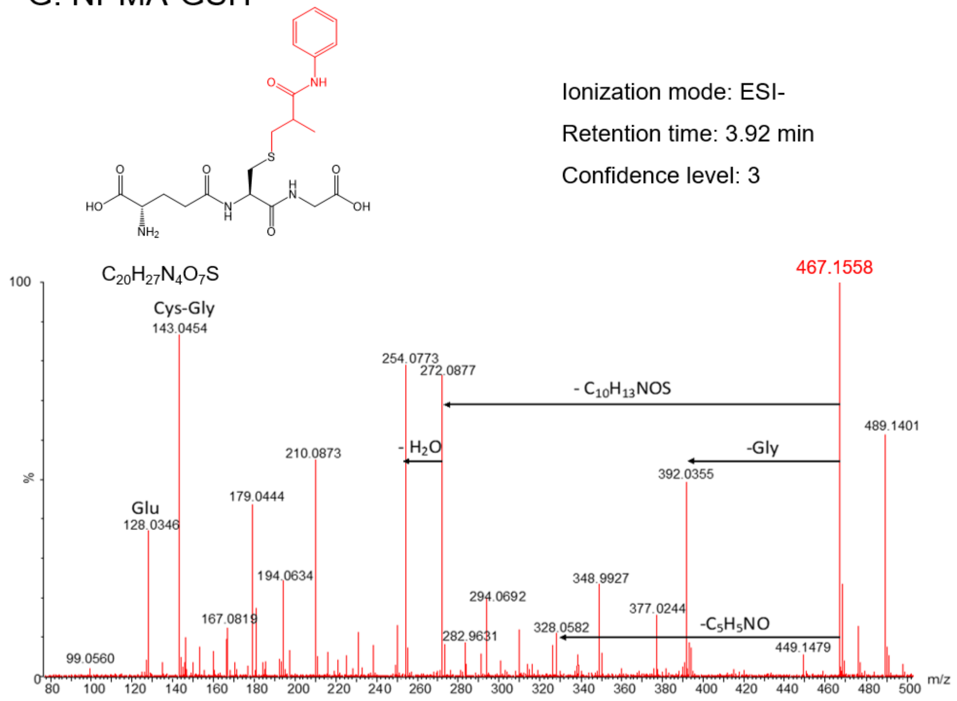

H: NPA-GSH

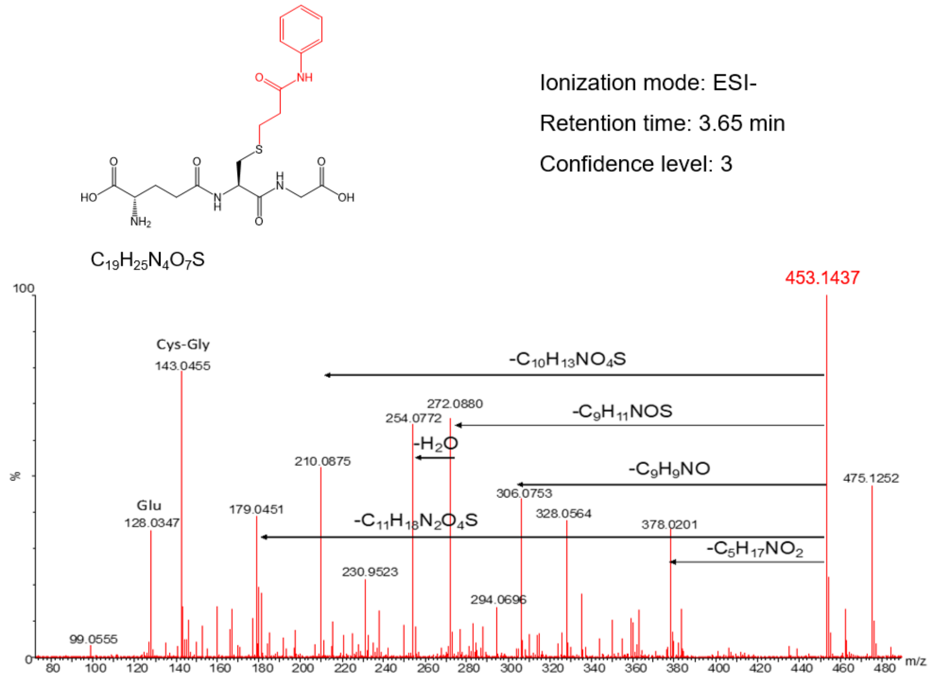

Figure S11: Structure, ionization mode, retention time, confidence level and MS/MS spectra of identified glutathione conjugates of acrylamides.

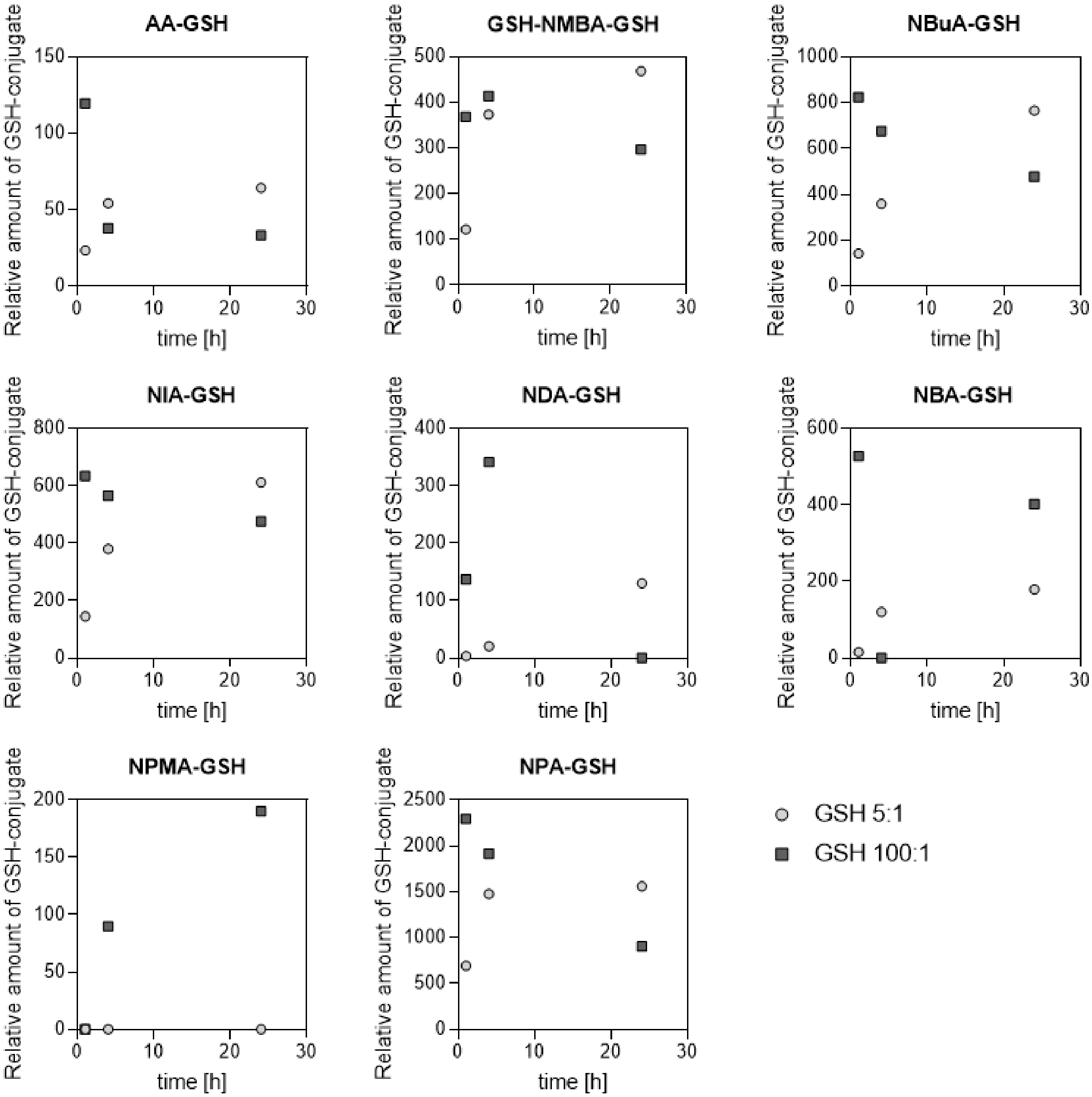

Figure S12: Relative amount of glutathione conjugates of acrylamides plotted against the incubation time. Light gray symbols indicate a GSH to acrylamide ratio of 5:1 and dark gray symbols indicate a GSH to acrylamide ratio of 100:1.

Table S6: Quantum chemical calculations of the test chemicals. The energy of the lowest unoccupied orbital (εLUMO) and the charge densities (q) of Cβ, Cα and C_1 were calculated.

Chemical	ε_{LUMO} (eV)	*q* (C_β)	*q* (C_α)	*q* (C_1)	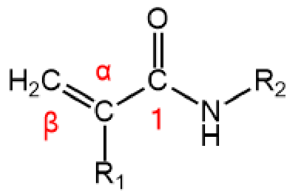
AA	0.10	-0.21	-0.22	0.57	
NMBA	0.09	-0.20	-0.22	0.56	
NMBA[a]	0.09	-0.21	-0.23	0.56	
NBuA	0.09	-0.21	-0.22	0.56	
NIA	0.10	-0.22	-0.21	0.55	
NDA	0.10	-0.21	-0.21	0.54	
MA	0.10	-0.24	-0.06	0.56	
NBA	0.09	-0.21	-0.22	0.56	
NPMA	0.08	-0.23	-0.07	0.55	
NPA	0.08	-0.21	-0.22	0.55	
NHMA	0.09	-0.23	-0.06	0.55	

[a]NMBA has two reactive groups, which were calculated separately.

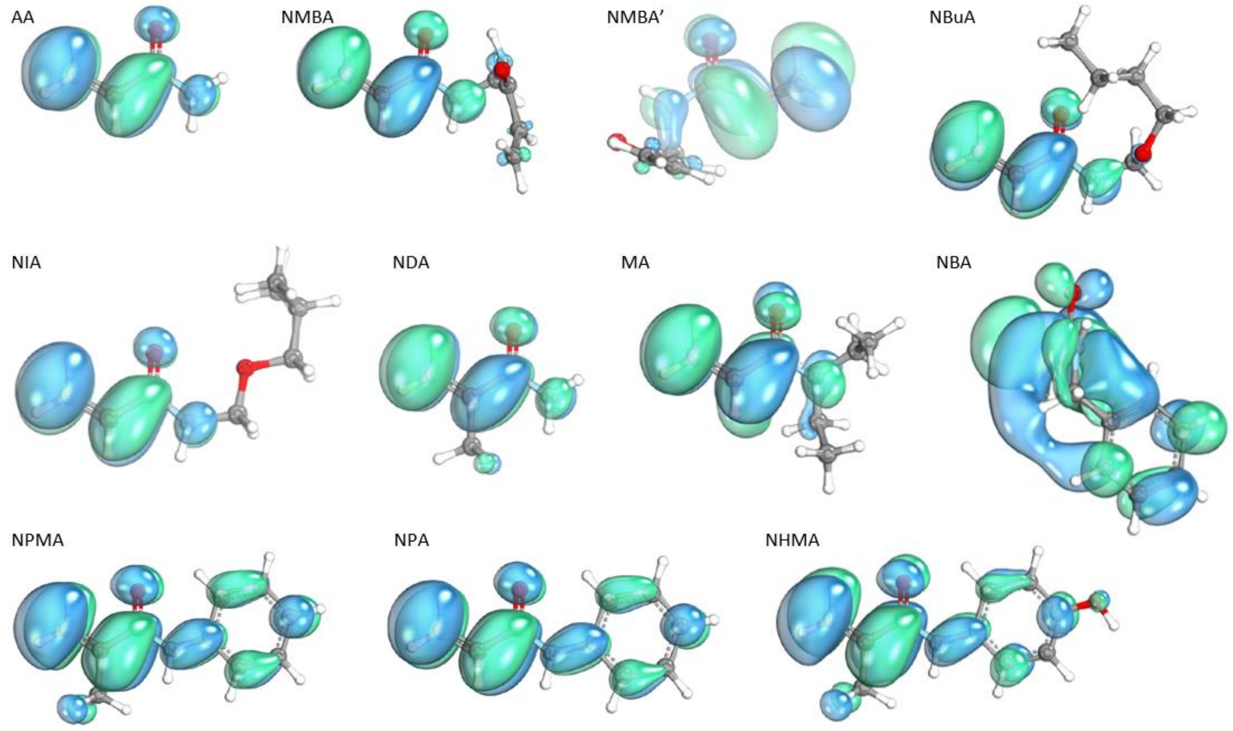

Figure S13: Lowest unoccupied molecular orbitals (LUMO) of the test chemicals.

References

(1) Ulrich, N., Endo, S., Brown, T. N., Watanabe, N., Bronner, G., Abraham, M. H., and Goss, K.-U. (2017) UFZ-LSER database v 3.2.1 [Internet], Leipzig, Germany, Helmholtz Centre for Environmental Research-UFZ. http://www.ufz.de/lserd.

(2) Henneberger, L., Mühlenbrink, M., Fischer, F. C., and Escher, B. I. (2019) C18-coated solid-phase microextraction fibers for the quantification of partitioning of organic acids to proteins, lipids, and cells. *Chem. Res. Toxicol. 32*, 168-178.
